# Developmental Emergence of Sparse Coding: A Dynamic Systems Approach

**DOI:** 10.1038/s41598-017-13468-z

**Published:** 2017-10-12

**Authors:** Vahid Rahmati, Knut Kirmse, Knut Holthoff, Lars Schwabe, Stefan J. Kiebel

**Affiliations:** 1 Department of Psychology, Technische Universität Dresden, 01187 Dresden, Germany; 2 Hans-Berger Department of Neurology, University Hospital Jena, 07747 Jena, Germany; 3 Department of Computer Science and Electrical Engineering, University of Rostock, 18059 Rostock, Germany

## Abstract

During neocortical development, network activity undergoes a dramatic transition from largely synchronized, so-called cluster activity, to a relatively sparse pattern around the time of eye-opening in rodents. Biophysical mechanisms underlying this sparsification phenomenon remain poorly understood. Here, we present a dynamic systems modeling study of a developing neural network that provides the first mechanistic insights into sparsification. We find that the rest state of immature networks is strongly affected by the dynamics of a transient, unstable state hidden in their firing activities, allowing these networks to either be silent or generate large cluster activity. We address how, and which, specific developmental changes in neuronal and synaptic parameters drive sparsification. We also reveal how these changes refine the information processing capabilities of an *in vivo* developing network, mainly by showing a developmental reduction in the instability of network’s firing activity, an effective availability of inhibition-stabilized states, and an emergence of spontaneous attractors and state transition mechanisms. Furthermore, we demonstrate the key role of GABAergic transmission and depressing glutamatergic synapses in governing the spatiotemporal evolution of cluster activity. These results, by providing a strong link between experimental observations and model behavior, suggest how adult sparse coding networks may emerge developmentally.

## Introduction

The proper development of neural networks is strongly activity-dependent^1^. A common feature of the immature cortex is the generation of synchronized network activity in which discrete events are separated by relatively long quiescent periods^2,3^. During development, this so-called cluster activity transitions to a sparse firing pattern as typically observed in adult networks^4^. Sparse firing is supposed to represent an efficient coding regime for processing and storing information in the mature cortex during adulthood^5,6^. The developmental transition from dense to sparse coding has been observed in all cortical areas examined to date^2,3,7^ and, in addition, in human cortex^8^. Moreover, since, unlike adult networks, eliciting large cluster activity is a ubiquitous feature of many immature neural structures^1,9^, sparsification is believed to be a universal phenomenon of neural network maturation. Although sparsification is thought to represent an essential aspect of cortical development, the mechanisms underlying this process are currently not understood. Data obtained from rodents led to the conclusion that the process of sparsification is largely, though not entirely^3^, independent of sensory inputs^2,7^. Strikingly, the time period of development during which sparsification occurs coincides with major changes in both intrinsic neuronal and synaptic properties. These include, for example, a profound decline in membrane resistance^2,10^, a steep increase in the number/density of both GABAergic and glutamatergic synapses^11,12^, an acceleration of the kinetics of postsynaptic currents^12^ as well as pronounced changes in short-term synaptic plasticity^10,12^.

Here, we describe a computational modelling approach based on experimentally measured trajectories of intrinsic neuronal and synaptic parameters in order to provide mechanistic insights into the generation of cluster activity and the transition from dense to sparse coding during development. To this end, we use the well-established extended Wilson-Cowan-type model accounting for mechanisms of short-term synaptic plasticity^13^. Despite the lack of well-established immature neuron models, as still many unspecified parameters need to be measured first, using this biophysically interpretable mean-field model enables the study of developing networks based on the average effects of these parameters at a network level. By combining this model with experimentally reported trajectories of neurobiologically plausible parameters during postnatal development, we found that we can emulate typically observed *in vivo* features of developing network function.

By using the mathematical tool of stability analysis, we derive three key results which help to better understand the sparsification process. Firstly, in the model we find that at early postnatal days, while the network is resting at its low-activity spontaneous state, an unstable state is built up in its firing activities. This is important, because we find that this transient, hidden unstable state is key to the emergence of large postnatal cluster activity. Using the model, we show how sparsification is driven developmentally, and how during this process the network’s information processing is refined. Secondly, we address the open question what mechanisms govern the spatiotemporal evolution of postnatal cluster activity. Thirdly, we quantify the effect of different maturational processes on sparsification. Together, this study provides the first mechanistic insights into the *in vivo* biophysical mechanisms underlying sparsification, and the implications of this process for the refinement of information processing.

## Results

**Figure 1:**
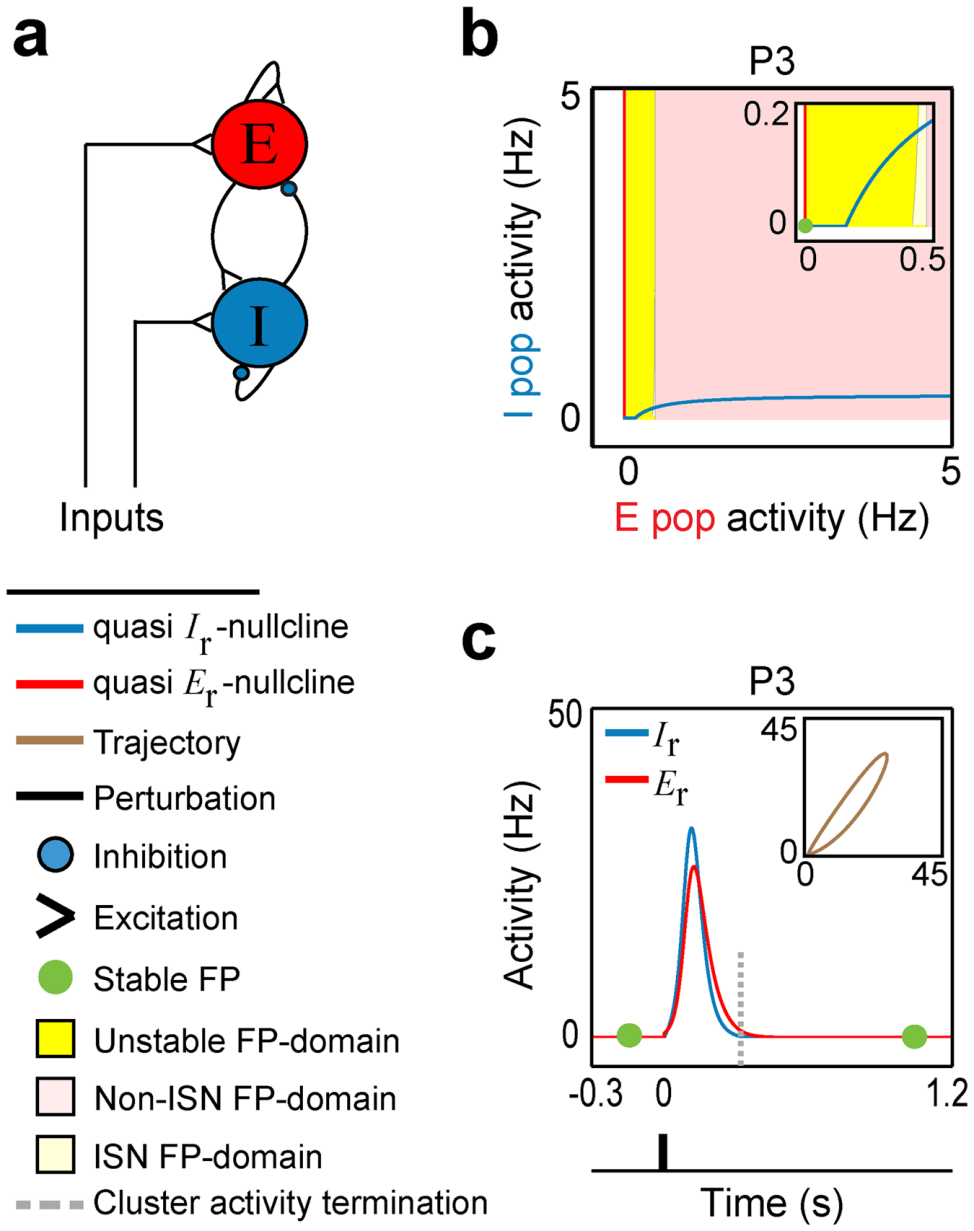
The stationary and cluster activity properties of a STP-RNN at P3. (**a**) Graph representing the spatially localized network of one excitatory, E, and one inhibitory, I, neuronal populations that are recurrently connected (RNN), and can receive inputs. (**b**) The *E*_r_-*I*_r_-plane of the RNN with dynamic synapses (STP-RNN) at postnatal day P3 (early period of physiological blindness). The colored regions show the fixed point (FP) domains of three possible operating regimes: unstable dynamics, inhibition-stabilized network (ISN), and Non-ISN; see Table 2 for details. (Inset) Zoom-in of the phase plane at lower activity ranges, overlaid by the FP of the STP-RNN. The ISN FP-domain is barely visible. The rest state and the vertical branch of the quasi ${E}_{{\rm{r}}}$ nullcline at ${E}_{{\rm{r}}}=0$ belong to the Non-ISN FP-domain. The horizontal branch of the quasi *I*_r_- nullcline at ${I}_{{\rm{r}}}=0$, forming the bottom-border of the ISN (barely visible) and unstable regimes, belong to the unstable FP-domain (see Supplementary Methods). Only the non-negative branches of the quasi *E*_r_- and *I*_r_- nullclines (i.e. in ≥0 Hz ranges) were displayed (e.g. see Supplementary Fig. 4, for the negative branches of the quasi nullclines). (**c**) Cluster activity triggered by an impulse perturbation (${e}_{{\rm{E}}}^{{\rm{per}}}$) to the E-population at time $t=0$ when the network was relaxed at the stable rest state. (Inset) Trajectories of cluster activity in *E*_r_-*I*_r_-plane. Simulations were performed for ${e}_{{\rm{E}}}^{{\rm{per}}}={e}_{{\rm{E}}}(t=0)=30$ Hz. For all simulations, parameter values can be found in Table 1.

To study developmental changes in postnatal neuronal activity, we used simulations of a spatially localized recurrent neural network with short-term synaptic plasticity (STP-RNN)^13^, where STP renders synaptic efficacies dynamic over time^14,15^; see Methods. This network is a mean-field Wilson-Cowan-type model of one excitatory (E) and one inhibitory (I) neuronal population with recurrent dynamic synaptic connections (Fig. 1a). This model has the advantage of being biophysically interpretable and mathematically accessible. The activity rates (*E*_r_ and *I*_r_) can be properly scaled to represent locally the average recorded activities in the populations. Here, we use experimentally reported, postnatal changes in neurobiologically plausible parameters (Table 1) to assess how the network’s spontaneous behavior and sensory processing properties are refined toward the postnatal onset of sensory transduction. To study the developmental states of visual cortical networks from before to after eye-opening, we selected four postnatal days (P) for modelling: P3 (period of physiological blindness), P10 (a few days before eye-opening), P14 (the day after eye-opening) and P20 (a few days after eye-opening)^3^. During this period, immature networks not only undergo the sparsification process but also a dramatic development in intrinsic neuronal and STP properties^10,12^; see Table 1 and Supplementary Methods. To derive the results, we used system dynamics methods described in the Methods section (see also Supplementary Methods). For quick reference, all important technical terms are explained in Table 2.

**Table 1:** Parameter values used in the developing STP-RNN model.

	P3	P10	P14	P20
${\tau }_{{\rm{E}}}$	0.045	0.030	0.020	0.010
${\tau }_{{\rm{I}}}$	0.0225	0.0150	0.010	0.005
${\tau }_{{{\rm{r}}}_{{\rm{E}}}}$	5.5	3	0.7	0.5
${\tau }_{{{\rm{r}}}_{{\rm{I}}}}$	5	2.5	0.4	0.2
${\tau }_{{{\rm{f}}}_{{\rm{E}}}}$	0.8	0.4	0.1	0.05
${\tau }_{{{\rm{f}}}_{{\rm{I}}}}$	0.8	0.4	0.1	0.05
${U}_{{\rm{E}}}$	0.9	0.8	0.65	0.55
${U}_{{\rm{I}}}$	0.9	0.8	0.55	0.4
${J}_{{\rm{E}}}$	3.7	7	6.3	5.5
${J}_{{\rm{I}}}$	0.1	3	4	4.5
***θ***_**E**_	0.3	0.47	0.7	1
***θ***_**I**_	0.3	0.5	1.7	2

This table lists the values of the developing STP-RNN parameters at postnatal days P3 (early period of physiological blindness), P10 (a few days before eye-opening), P14 (the day after eye-opening) and P20 (a few days after eye-opening). The time constants are in units of [s]. All parameters are consistent across the figures, unless stated otherwise. ${\tau }_{{\rm{i}}}$ is an approximation to the decay time constant of postsynaptic responses, ${\tau }_{{{\rm{r}}}_{{\rm{i}}}}$ is the synaptic recovery time constant of depression, ${\tau }_{{{\rm{f}}}_{{\rm{i}}}}$ is the synaptic facilitation time constant, ${U}_{{\rm{i}}}$ is the release probability, ${J}_{{\rm{i}}}$ is the absolute synaptic efficacy, and *θ*_i_ is the population activity threshold (in units of [Hz]). The quantities express the mean parameter values for excitatory (${\rm{i}}={\rm{E}}$) and inhibitory (${\rm{i}}={\rm{I}}$) populations. Although not listed here, for simplicity, we fixed ${G}_{i}$ (the linear input-output gain above *θ*_i_) at 1, while considering its developmental changes in values of ${J}_{{\rm{i}}}$. See “Parameterization of postnatal developing networks” section in Supplementary Methods for the list of experimental papers that we used to determine these developmental changes in parameter values.

**Table 2:** Overview of technical terms and analysis metrics.

Abbreviation	Description
RNN	Recurrent neural network (RNN) of one E-I pair of synaptically coupled populations*.
STP-RNN & Static-RNN	A RNN composed of synaptic connections with short-term plasticity (STP-RNN) which renders the synaptic efficacies dynamic over time. The RNN with constant efficacies is a Static-RNN^†^.
Frozen STP-RNN	A STP-RNN with the synaptic efficacies frozen e.g. at the FP; thus, a Static-RNN-type model^$^.
*E*_r_-*I*_r_-plane	A 2D phase plane of E- and I-population activity rates ($${I}_{{\rm{r}}}$$ vs. $${E}_{{\rm{r}}}$$)^‡^.
FP	A fixed point (FP) is the steady state of a network, and is determined as the intersection of (quasi) *E*_r_- and *I*_r_- nullclines in the *E*_r_-*I*_r_-plane^‡^.
Spontaneous activity	Network activity in the absence of stimulus (or sensory input)*.
Attractor	A non-quiescent stable FP (in this paper); also known as memory or persistent activity state.
Attraction domain	For a stable FP (in this paper), a region in phase plane comprising all initial conditions that lead to that FP.
Amplification-threshold	The outer border of the attraction domain of the rest state in the Frozen STP-RNN, beyond which network perturbations undergo an overall continuous growing (in the Frozen STP-RNN) or will effectively trigger cluster activity (in the STP-RNN).
Nullcline	For example, an excitatory nullcline is a set of points (a curve) in *E*_r_-*I*_r_-plane for which $$d{E}_{{\rm{r}}}/dt=0$$ ^‡^.
FP-domain	For example, an unstable FP-domain is a domain of all potential FPs in the *E*_r_-*I*_r_-plane, at which the network will be unstable^¶^.
$$AOD$$	Area of domain (AOD); for example, *AOD*_Unstable_ is the area of the unstable FP-domain^¶^.
ISN	Inhibition-stabilized network (vs. Non-ISN) which requires sufficiently strong, dynamic inhibitory feedback to preserve its overall stability.§
*AOD*_ISN/Unstable_	=*AOD*_ISN_/*AOD*_Unstable_; this ratio is used to measure the relative area of the ISN FP-domain vs. unstable FP-domain^¶^.
PS	The near coincident firing of many neurons is often referred to as population spike (PS)^#^.
Cluster activity	Network spike (PS_net_), which involves both E- and I-populations; i.e. both PS_E_ and PS_I_^#^.
$$P{S}_{{\rm{net}}}^{{\rm{amp}}}$$	The scaled amplitude of PS_net_, which provides a qualitative approximation to the cluster activity size^#^.

This table lists the descriptions for abbreviations and definitions which we used in our study. The sections in Supplementary Methods providing detailed descriptions: *Model description, ^†^STP-RNN, ^$^Frozen STP-RNN, ^‡^Computation of 2D phase planes, ^¶^Operational FP-domains, ^§^Characterization of operating regimes, and ^#^Population spike.

### Transient unstable state hidden in firing activities

We first address what mechanisms may underlie the emergence of cluster activity in immature networks, by analyzing model behavior at P3 as a representative early postnatal stage.

Previous experimental^16,17^ and modeling studies^18,19^ usually considered adult networks to be bi- or multi-stable where, e.g., spontaneous (stimulus-absent) network activity can transition between two stable activity states: A relatively quiet activity state, and a higher activity state. For early postnatal stages, our stability analysis and simulations indicate that the proposed developing network model is actually mono-stable whose only stable state is a quiescent, spontaneous state (Fig. 1b). To demonstrate this, we plotted in Fig. 1b the so-called phase plane of network dynamics in terms of the average activity in the E- and I-populations (*E*_r_-*I*_r_ **-**plane; Table 2). Figure 1b shows that the network’s fixed point (FP; Table 2) is located at *E*_r_ = *I*_r_ **=** 0 which is essentially a stable activity state of the network (see Supplementary Methods). In the following, we will call this specific FP the rest state of the network. This analysis result fits well with experimental findings of near-zero hertz spontaneous activity of immature populations^3,20^, and the absence of spontaneous network’s persistent activity states mainly during periods of physiological blindness^7,21^.

How can a quiescent, immature, mono-stable network generate large cluster activity? By analyzing network perturbations, we found strikingly that while a P3 network model was relaxed at its stable rest state, threshold-crossing perturbations of E-population were amplified profoundly (Fig. 1c, Supplementary Fig. 1c). This amplification resulted in population spikes (PSs, Table 2), which, in the model, we assume is the analogue to experimentally observed cluster activity. Note that this perturbation can be due to, e.g., a single-shock electrical stimulation^22^, the onset of a longer-lasting external input (Supplementary Fig. 2c), or a random input driven by spontaneous retinal waves or the thalamus^3,20^.

**Figure 2:**
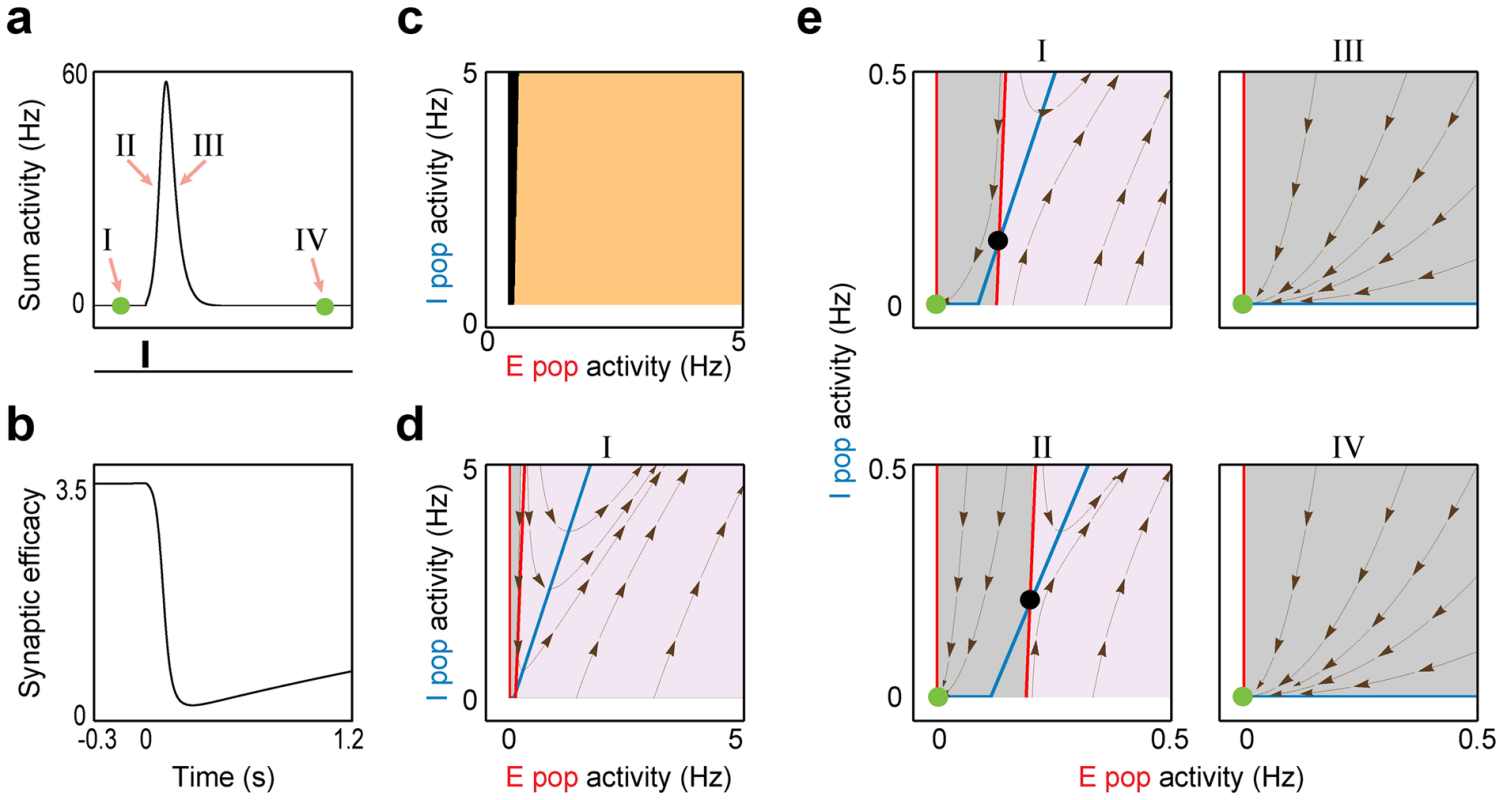
The transient unstable state hidden in fast (i.e. firing activity) dynamics of a STP-RNN at P3. (**a**) The same cluster activity as in Fig. 1c, but shown in terms of the sum activity ${A}_{{\rm{sum}}}={E}_{{\rm{r}}}+{I}_{{\rm{r}}}$. (**b**) The time-evolution of synaptic efficacy of the recurrent excitatory connection ${J}_{{\rm{E}}}\,{x}_{{\rm{E}}}\,{u}_{{\rm{E}}}$, during the network activity shown in (**a**). (**c**) Activity-perturbation domains of STP-RNN. For the network relaxed at the rest state, setting the initial condition of the network activity at different E-and I-activity values revealed two different types of domains; amplification domain (cream-colored region): After perturbation, the sum activity was effectively amplified and cluster activity emerged; Non-amplification domain (black region): After perturbation, the sum activity monotonically decayed back to the rest state. Note, for the P3 network (mono-stable), both these domains are attraction domains of the rest state in the STP-RNN. (**d**) The *E*_r_-*I*_r_-plane and the *E*_r_- (red) and *I*_r_- nullclines (blue) of the STP-RNN with frozen synaptic efficacies (i.e. Frozen STP-RNN) at the rest state (see the time I in (**a**); $t=-150$ ms, relative to onset of input). Grey region: The attraction domain of the stable rest state in the Frozen STP-RNN. Purple region: The activities initiated here undergo an overall continuous growing (non-attraction domain). We call the border between these two regions the amplification-threshold. This border is approximately the same as the border between the two domains in (**c**). (**e**) Disappearance of the hidden unstable state during cluster activity. (I): Zoom-in of (**d**) at lower activity rates, overlaid by the FPs of the corresponding Frozen-RNN. (II-IV): Same as (I), but for synaptic efficacies frozen at different sample times (see (**a**)): (II): $t=75$ ms, (III): $t=175$ ms, (IV): $t=1000$ ms. Black dots show the unstable FPs. Dark-brown streamlines show, at each point, the local direction of sample trajectories in the corresponding Frozen-RNN.

While previous studies showed the underlying mechanism of PSs generation mainly in adult bi-stable network models^18,19^, here we sought to address this mechanism in an immature mono-stable network (Fig. 2). As the initial phase of PSs is known to be governed by the network’s fast (i.e. firing activity) dynamics^18^, we re-plotted the *E*_r_-*I*_r_-plane after freezing the network’s slow (i.e. STP) dynamics at the rest state (Fig. 2d); thus, it is turned to a Static-RNN (Table 2) with frozen synaptic efficacies (Frozen STP-RNN). Surprisingly, we found that in addition to the stable rest state, there is an unstable FP hidden in the network’s fast dynamics (i.e. in the respective Frozen STP-RNN, Fig. 2e), which does not exist in the (non-frozen) STP-RNN (Fig. 1b). This unstable FP (black dot, Fig. 2e), which is located close to the rest state, can confine the attraction domain (grey region, Table 2) of the stable rest state in the *E*_r_-*I*_r_-plane of the corresponding Frozen STP-RNN (Fig. 2d); but, clearly, not in the *E*_r_-*I*_r_-plane of the STP-RNN itself (Fig. 2c). We call the outer border of this domain in Fig. 2d (before perturbation) the amplification-threshold. Almost exactly the same border exists for activity-perturbation domains of the STP-RNN (Fig. 2c). Cluster activity is initiated by an activity perturbation exceeding this threshold, where the activity will be moved far from the rest state in both corresponding networks with frozen (Frozen STP-RNN) or dynamic (STP-RNN) synapses. Strikingly, when freezing synaptic efficacies at different times during a cluster activity (Fig. 2a), we can see that this unstable FP disappears roughly after the peak of cluster activity (Fig. 2e), thereby allowing network activity to converge back to its stable rest state in the STP-RNN (Fig. 2a). This disappearance is mainly because of the weakening of the excitatory synapses at high activity rates (Fig. 2b); these synapses provide positive feedback to the network. Importantly, as it can be seen in Fig. 2e (panel IV), while the network is again at the origin after cluster activity, sufficient recovery time is required for the re-emergence of the unstable FP.

Our analyses further show that the experimentally observed variability in size (i.e. the number of network neurons recruited by cluster activity) and duration of spontaneous cluster activities at each postnatal day^2,3^ is effectively governed by the relatively long recovery time of immature excitatory synapses (Supplementary Fig. 1). In addition, in our model, cluster activity was generated only if the perturbation strength was sufficiently large (Supplementary Fig. 1c and d) and was able to push network activity beyond the amplification-threshold (Fig. 2c and d). This is consistent with experimental reports of immature networks^7,23^, and may underlie a relatively all-or-none characteristic of postnatal cluster activity.

### The route towards sensory processing

The sparsification of activity patterns sets in around eye-opening^3,7^. We now use our model at the stages P10 to P20 to address the question what refinements of the developing network enable sparsification.

**Figure 3:**
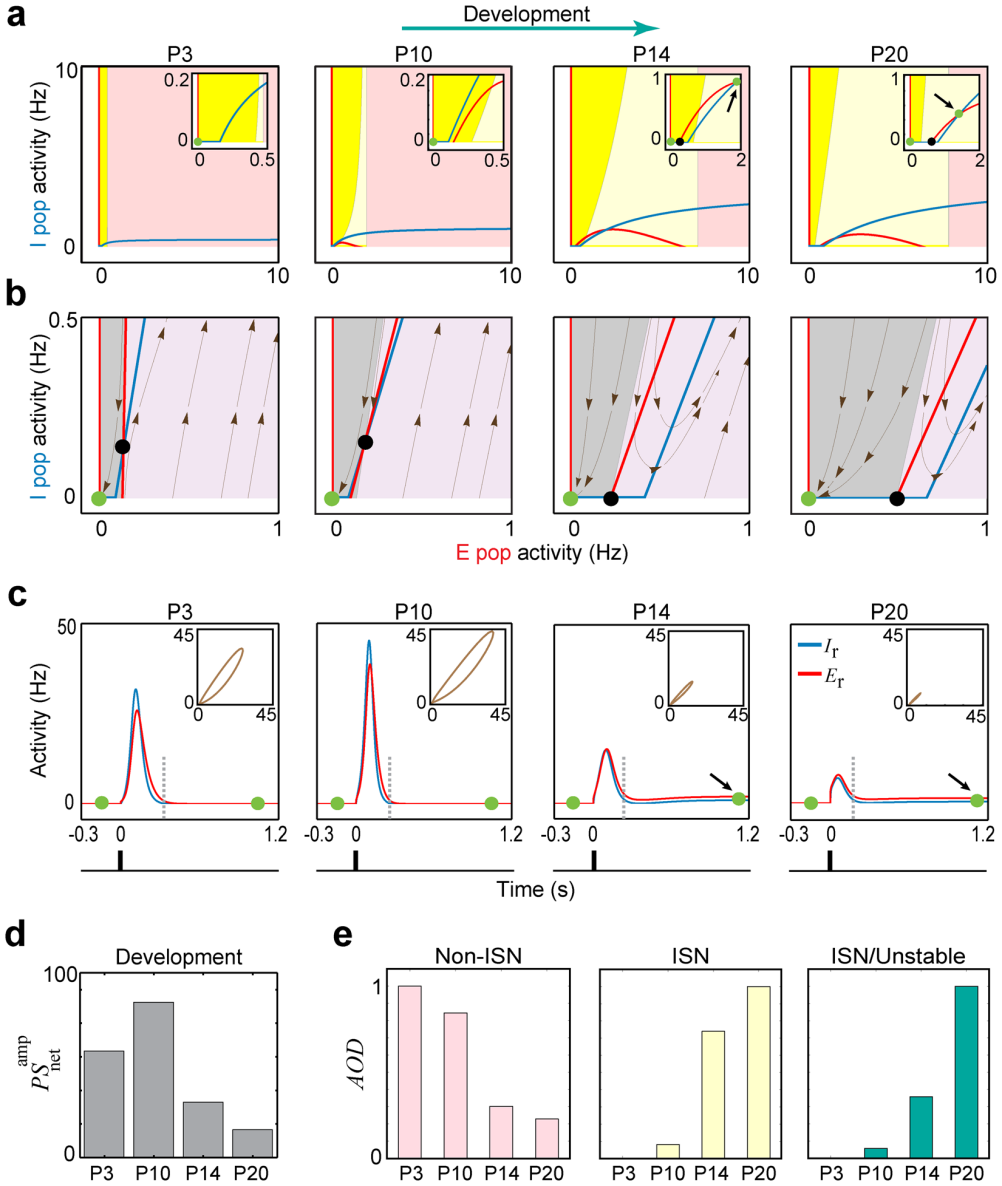
Sparsification process in developing cortex. (**a**) Postnatal changes of the network’s stationary firing dynamics. The same format is used as in Fig. 1b; unstable dynamics (yellow region), ISN (light-yellow region), and Non-ISN (pink region). (**b**) The existence of a hidden unstable state (black dot) in the firing activity of developing networks. The same format is used as in Fig. 2e, panel I. The networks were frozen at the rest state ($t=-150$ ms). (**c**) Postnatal changes of the network’s transient firing dynamics where cluster activity was triggered by an impulse perturbation. The same format is used as in Fig. 1c. P3: early period of physiological blindness, P10: a few days before eye-opening, P14: the day after eye-opening, and P20: a few days after eye-opening. Green dots represent the stable FPs. (**d**) Developmental changes in the size of cluster activities, estimated qualitatively by $P{S}_{{\rm{net}}}^{{\rm{amp}}}$. (**e**) Developmental changes in the area of the FP-domain (*AOD*) of Non-ISN and ISN operating regimes, and the ratio *AOD*_ISN/Unstable_ of areas of ISN and unstable FP-domains. The developmental increase in *AOD*_ISN/Unstable_ indicates that the unstable FP-domain was effectively decreased, and replaced by the ISN FP-domain. For each panel, the values were normalized to the maximum value during all four stages. At each of these stages, the *AOD* s were computed based on the square *E*_r_-*I*_r_-plane with the lower-left corner located at the rest state (origin) and the upper-right corner (i.e. $[{E}_{{\rm{r}}}^{{\rm{\max }}},\,{I}_{{\rm{r}}}^{{\rm{\max }}}]$) at [10,10] Hz. Moreover, the developmental decrease in $AO{D}_{\mathrm{Non}-\mathrm{ISN}}$ just means that the E-activity rate after which the Non-ISN FP-domain starts in the *E*_r_-*I*_r_-plane, was shifted to higher levels. See Table 2 for details about technical terms.

Firstly, similarly as for P3, at all later stages up to P20 in the model we found that for a developing network relaxed at its stable rest state (Fig. 3a), again an unstable FP is hidden in the network’s fast (i.e. firing activity) dynamics (Fig. 3b). Consequently, a threshold-crossing perturbation of the E-population at rest state led to cluster activity (Fig. 3c). In addition, our analysis revealed that the amplification-threshold tends to move toward higher E-activity rates, from P3 to P20 (Fig. 3b, Supplementary Fig. 3). This result suggests that a stronger input may be required to trigger (large) cluster activities at late development stages.

Experimental *in vivo* findings show that the cluster activity size starts to drop dramatically around the end of the second postnatal week^2,3^. Here, we consider this developmental decrease in cluster activity size as an indicator for the developmental transitioning from dense to sparse coding (sparsification process); similarly to experimental studies^2,3^. In our model, we approximate the cluster activity size by a measure of the average activity of both E- and I-populations ($P{S}_{{\rm{net}}}^{{\rm{amp}}}$; see Table 2 and Methods). Using this measure, we observed, similarly as in experiments, that cluster activity size undergoes developmental reduction between P10 to P20 (Fig. 3c and d, see also Supplementary Fig. 2c): It was relatively large at P10 ($P{S}_{{\rm{net}}}^{{\rm{amp}}}\approx 85$), but starkly reduced after eye-opening (P14; $P{S}_{{\rm{net}}}^{{\rm{amp}}}\approx 30$), followed by some further reduction to P20 ($P{S}_{{\rm{net}}}^{{\rm{amp}}}\approx 15$).

Secondly, the results also show that immature networks probably lack any spontaneous, non-quiescent stable FP (i.e. a spontaneous attractor or persistent activity state) up to a few days prior to eye-opening. In Fig. 3a this is indicated by the existence of only one green dot at P3 and P10. However, after eye-opening, new spontaneous FPs with higher activity rates than the rest state emerged (note the non-origin FPs at P14 and P20 in Fig. 3a). This is compatible with experimental data showing the existence of spontaneous persistent cortical activity for stages mostly after the second postnatal week^3,21,24^ and their absence during early development^7,21^. In our model, in the absence of a stimulus, these FPs emerge mainly because of the developmental increase and decrease in background activity^2,3,7^ and membrane resistance^2,10^, respectively. Their combined effects determine the increase in population activity thresholds *θ*_E_ and *θ*_I_ (see also Supplementary Fig. 4). While in the model the network’s FP domains (FP-domains; Table 2) are not affected by these thresholds (see “Characterization of operating regimes” section in Supplementary Methods and also Supplementary equation (17)), the developmental reduction in the network instability (yellow regions, Fig. 3a) allows for these FPs to be stable. This means that attractors start emerging, under a wide range of E-activity; note the non-origin green dots at P14 and P20 in Fig. 3a.

Thirdly, the emergence of spontaneous attractors not only turns the developing mono-stable network (at P3 and P10) into a bi-stable (at P14 and P20) network (Fig. 3a), but also has a striking effect on the dynamical trajectory of cluster activity. That is, in the model, we found a fundamental difference between spontaneous postnatal cluster activities and those in more mature networks (Fig. 3c): before eye-opening (P3 and P10), the cluster activity is of the so-called mono-stable type (see Supplementary Methods), while after eye-opening the cluster activity can be also of the bi-stable type. The difference is that a mono-stable cluster activity (P3 and P10 in Fig. 3c) is initiated and terminated at the stable rest state, while a so-called bi-stable cluster activity is initiated at the stable rest state but converges to a spontaneous attractor (P14 and P20 in Fig. 3c, green dots). This difference may be interpreted as a first expression of information processing where the newly emerged attractors, which a bi-stable cluster activity converges to, can be seen as representative states that are the basis of perception. In the model, the time required for an effective transition to the attractor, and thus an effective representation of perceptual stimuli, is probably equal to the duration of cluster activity (see dashed grey lines, Fig. 3c): ~240 ms at P14 and ~180 ms at P20; these transition times are consistent with experimental observations at these stages^17,24^.

Moreover, our simulations showed that the spatiotemporal characteristics (i.e. size and duration) of mono-stable cluster activities are in general considerably more robust to interfering perturbations (P3 and P10, Supplementary Fig. 5), as compared to the bi-stable ones (P14 and P20, Supplementary Fig. 5). This suggests that the trajectories underlying mono-stable cluster activities (P3 and P10) can be seen as signals with high signal-to-noise ratio, which may act as a reliable neuronal communication mechanism in neonatal networks. The decreased robustness of bi-stable cluster activities after eye-opening (P14 and P20) can in principle augment the network’s information processing capability by making it more flexible in responding to sensory input.

In addition, we found that at the new spontaneous attractors (non-origin green dots; Fig. 3a) the network operates as a so-called inhibition-stabilized network (ISN, light-yellow regions, see also Table 2)^25,26^. An ISN regime is thought to allow the cortex to process complex computations^27^, and some experimental evidence indicates that adult cortical networks operate as ISNs^25,28^. Our results show that the ISN regime becomes effectively accessible after eye-opening, as the unstable FP-domain, which is confined to low E-activity ranges, is developmentally substituted by an ISN FP-domain. This indicates a developmental increase in the ratio of areas of the ISN (*AOD*_ISN_) and unstable FP-domains (*AOD*_Unstable_), which we quantified by *AOD*_ISN/Unstable_ (Table 2) in Fig. 3e. In practice, this means that in parallel to the strengthening of the sensory inputs after eye-opening, a larger set of potential stimulus-evoked ISN attractors will be accessible for the network, presumably to perform complex sensory computations.

Overall in our model, the process of sparsification is translated not only to a potent reduction of postnatal network instability but also to a potential emergence of new attractors and information processing capabilities.

### Spatiotemporal evolution of postnatal cluster activity

What mechanisms govern the spatiotemporal evolution (i.e., size and duration) of cluster activity during development *in vivo*? We addressed this question by analyzing how a postnatal cluster activity, once on a trajectory away from the rest state, is brought back to either the rest state or another FP with an activity level higher than the rest state.

In our model, there are two important factors: (i) the inhibitory transmission, which is believed to play a critical role in the stabilization of adult networks^25,28^, and (ii) STP, which can dynamically control the gain of neuronal responses^14^ and acts strongly depressing in immature networks^10,12^.

**Figure 4:**
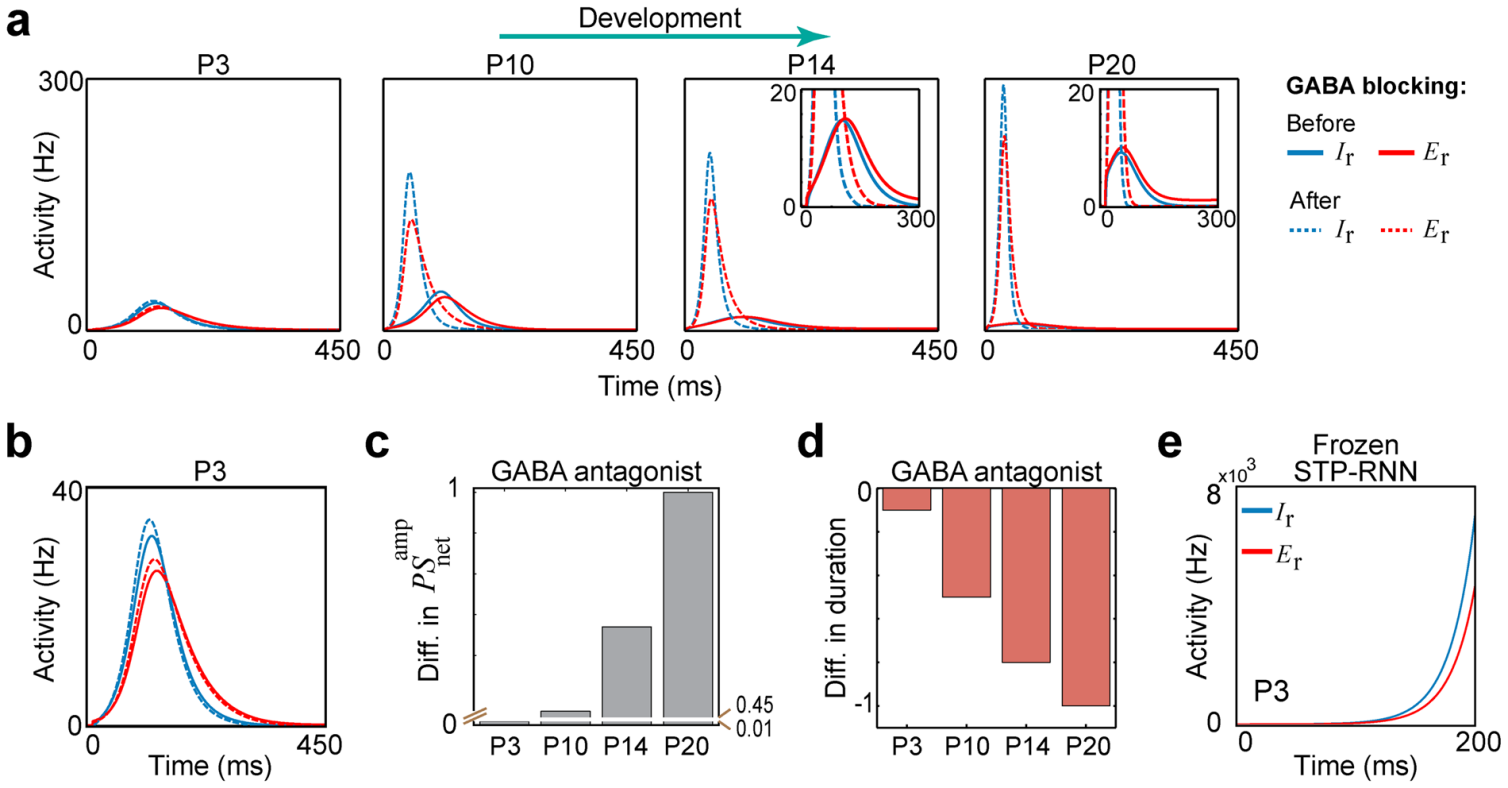
Contribution of GABAergic transmission to cluster activity during course of development. (**a**) The effect of blocking GABAergic receptors (i.e. ${J}_{{\rm{I}}}=0$) on cluster activities, during development. The plots show that even with this blockage, cluster activity that emerges from the rest state can still converge back to this state. Solid lines: cluster activity before the blockage, Dashed lines: cluster activity after the blockage. (**b**) Zoom-in of P3 in (**a**) at lower activity rates. Note the increase in E- and I-activities after the blockage. (**c**) Normalized differences in cluster activity size before and after the blockage (*after* minus *before*), at the four developmental stages. The values were normalized to the maximum difference observed throughout P3 to P20. (**d**) Same as (**c**), but for cluster activity duration. (**e**) The effect of freezing the synaptic efficacies at the stable rest state, at P3. A threshold-crossing perturbation (see light-purple region in Fig. 2d) leads to run away activities; note the scale of the y-axis.

Firstly, we found that cluster activity initiated at the rest state can still converge back to this state even when blocking GABAergic receptors, at all stages from P3 to P20 (Fig. 4a). This finding suggests that inhibitory processing may not be necessary for stability in the developing network with depressing excitatory synapses. However, blockage of GABAergic receptors yielded an increase in cluster activity size (Fig. 4a and c), consistent with *in vivo* reports^20,29^. This blockage effect on cluster activity size became more pronounced during the course of development (Fig. 4c), in spite of the developmental reduction in the network instability (e.g. see *AOD*_ISN/Unstable_ in Fig. 3e). This finding may underlie a developmental enhancement of an effective contribution of inhibitory transmission to network activity (see also Supplementary Fig. 2d showing the ability of inhibitory inputs to turn off activity at the attractor^22^, after eye-opening). In addition, we found that the blockage of GABAergic receptors shortened the cluster activity duration, for example, from approximately 330 to 320 ms at P3 (Fig. 4a and b) and from approximately 265 to 210 ms at P10 (Fig. 4a). The range of these cluster activity durations in our model is compatible with previous experimental reports^7,30^. Moreover, we found that this blockage effect on cluster activity duration increased from P3 to P20 (Fig. 4d).

Secondly, in contrast to GABAergic transmission, we found that at P3 the simulated removal of the STP effect led to run-away excitation (Fig. 4e). After freezing the synaptic efficacies (see Supplementary Methods) at the stable rest state, a threshold-crossing perturbation of the E-population (similarly as in Fig. 1c) caused a seizure-like surge of network activity (Fig. 4e). Importantly, this means that the rest state in the corresponding Frozen STP-RNN is a locally, but not globally, stable FP (see Methods). Similar results were observed for P10 to P20 (data not shown). Accordingly, we conclude that the strongly depressing immature STP, but not inhibitory synaptic transmission, guarantees the stabilization of postnatal cluster activities during the course of development. This finding also holds, when GABAergic transmission is considered as excitatory at the network level during the first postnatal week (see Supplementary Methods and Supplementary Fig. 6b). Under this assumption, blocking GABAergic receptors led to a decrease in cluster activity size, which is not compatible with *in vivo* data^20,29^. Therefore, our modeling results (Fig. 4b) are in agreement with recent *in vivo* findings that while GABA acts as a mainly depolarizing transmitter during the first postnatal week, it exerts an inhibitory effect on the underlying network activity in the immature cortex and hippocampus^20,31^.

Moreover, cluster activity was abolished by simulated blockage of glutamatergic receptors (data not shown), consistent with experimental *in vivo* data^20,29^. This is because the main source of the network instability in our model is determined by glutamatergic synapses which provide positive feedback to the network. Therefore, blocking glutamatergic receptors removed all unstable FPs as well as the attractors in Fig. 3a and forced spontaneous network activity to the rest state (as observed experimentally^17,22^), and removed the unstable FP hidden in the network’s fast dynamics in Fig. 3b, throughout P3 to P20 (data not shown). In addition, note that the GABAergic transmission can play an important modulatory effect on this instability (see Supplementary Methods), where an increase in the efficacies of inhibitory synapses can effectively attenuate the instability (not shown). This implies that relatively weak GABAergic inhibition (Table 1) is permissive for the generation of the unstable states (e.g. see Fig. 3a and b) in developing networks; note that in our model these synapses strengthen during development (Table 1).

In sum, we found that strongly depressing excitatory synapses have a key role in the termination of postnatal cluster activities, whereas GABAergic transmission mainly regulates their spatiotemporal evolution.

### Key maturational processes mediating sparsification

The most influential, maturational processes mediating sparsification are still not clearly understood^2,3^. By using our modelling approach, we mechanistically quantified the impact of different network parameters on the emergence of sparse coding.

**Figure 5:**
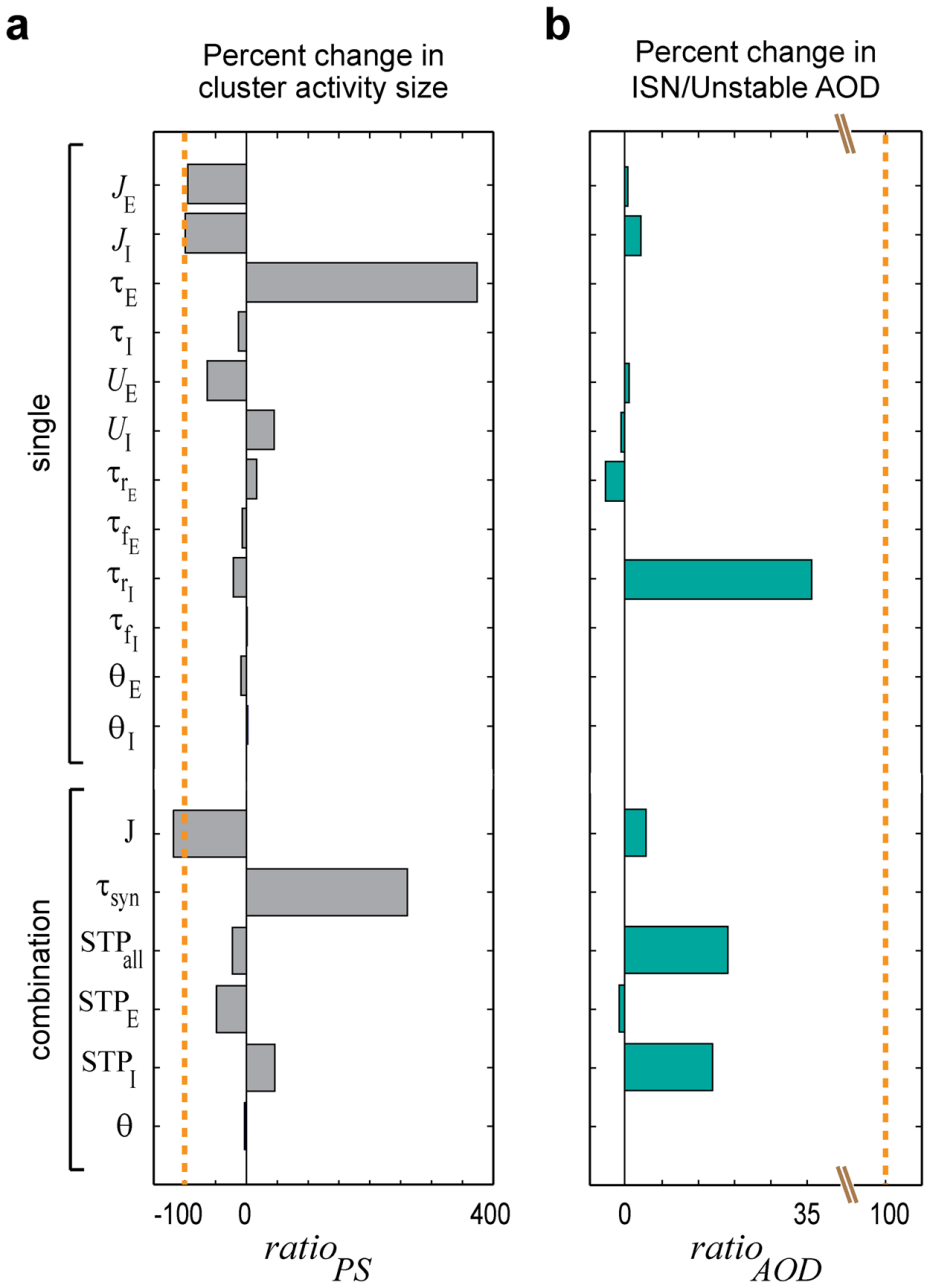
Impact of specific maturational processes on the sparsification process. We measured the change in network behavior when, virtually, we only mature a single parameter or small sets of similar parameters from P10 to P20. This enables us to indicate key parameters required for sparsification. (**a**) The plotted values of $rati{o}_{PS}$ measure the modification-induced changes in $P{S}_{{\rm{net}}}^{{\rm{amp}}}$, i.e. the size of simulated cluster activity, relative to the decrease when transitioning from P10 to P20. The dashed orange line at $rati{o}_{PS}=-100\, \%$ indicates the normal amount of decrease in $P{S}_{{\rm{net}}}^{{\rm{amp}}}$, as expected when transitioning from P10 to P20. (**b**) The plotted values of $rati{o}_{AOD}$ measure the modification-induced change in $AO{D}_{{\rm{ISN}}/{\rm{Unstable}}}$, i.e. the ratio of areas of ISN and unstable FP-domains, relative to the increase when transitioning from P10 to P20. The dashed orange line at $rati{o}_{AOD}=+100\, \%$ indicates the normal amount of increase in *AOD*_ISN/Unstable_, as expected when transitioning from P10 to P20. See Methods for the formulas of $rati{o}_{PS}$ and $rati{o}_{AOD}$. For computing *AOD*_ISN/Unstable_, we considered the *E*_r_-*I*_r_-plane plots with $[{E}_{{\rm{r}}}^{{\rm{\max }}},\,{I}_{{\rm{r}}}^{{\rm{\max }}}]$ = [10,10] Hz. Parameters combinations are ${\rm{J}}=\{{J}_{{\rm{E}}},{J}_{{\rm{I}}}\}$, ${{\rm{\tau }}}_{{\rm{syn}}}=\{{\tau }_{{\rm{E}}},{\tau }_{{\rm{I}}}\}$, ${{\rm{STP}}}_{{\rm{all}}}=\{{{\rm{STP}}}_{{\rm{E}}},{{\rm{STP}}}_{{\rm{I}}}\}$, ${{\rm{STP}}}_{{\rm{E}}}=\{{U}_{{\rm{E}}},{\tau }_{{{\rm{r}}}_{{\rm{E}}}},{\tau }_{{{\rm{f}}}_{{\rm{E}}}}\}$, ${{\rm{STP}}}_{{\rm{I}}}=\{{U}_{{\rm{I}}},{\tau }_{{{\rm{r}}}_{{\rm{I}}}},{\tau }_{{{\rm{f}}}_{{\rm{I}}}}\}$, ${\rm{\theta }}=\{{\theta }_{{\rm{E}}},{\theta }_{{\rm{I}}}\}$.

To do this, we replaced single parameters or parameter combinations of the P10 network model by their respective values at P20 and measured to what extent this modification can account for the normal decrease in $P{S}_{{\rm{net}}}^{{\rm{amp}}}$ and normal increase in *AOD*_ISN/Unstable_, when transitioning from P10 to P20. To quantify effects we computed $rati{o}_{PS}$ and $rati{o}_{AOD}$ as the ratios of the modification-induced changes in $P{S}_{{\rm{net}}}^{{\rm{amp}}}$ and *AOD*_ISN/Unstable_, relative to their respective normal developmental changes during this period (Fig. 5). We found that only three parameters caused a substantial decrease in $P{S}_{{\rm{net}}}^{{\rm{amp}}}$ (Fig. 5a): the two absolute synaptic efficacies (${J}_{{\rm{E}}}$ and ${J}_{{\rm{I}}}$) caused the largest decrease close to that in the normal transition and, to a minor degree, the excitatory release probability ${U}_{{\rm{E}}}$. Strikingly, for the excitatory synaptic time constant (${\tau }_{{\rm{E}}}$) the modification led to an increase in $P{S}_{{\rm{net}}}^{{\rm{amp}}}$, i.e. when this parameter is changed on its own, it tended to reverse sparsification (see also Supplementary Figs 6c and 7). We obtained qualitatively similar conclusions when analyzing the effect of replacing single parameters or parameter combinations of the P20 network model by their values at P10 (Supplementary Fig. 8a). When testing for changes in *AOD*_ISN/Unstable_, we found that the inhibitory synaptic depression time constant ${\tau }_{{{\rm{r}}}_{{\rm{I}}}}$ had the strongest contribution to the normal transition from P10 to P20, whereas all other single parameter substitutions had relatively weak effects (Fig. 5b). Note that the change in ${\tau }_{{{\rm{r}}}_{{\rm{I}}}}$ appears to be a necessary but not a sufficient condition for the normal transition from P10 to P20, as substituting ${J}_{{\rm{E}}}$ and ${J}_{{\rm{I}}}$ in the P20 model by their respective P10 values virtually abolished the developmental change in *AOD*_ISN/Unstable_ (Supplementary Fig. 8b). Figure 5 further shows that the modification effect of the population activity thresholds (*θ*_E_ and *θ*_I_) was virtually negligible for $rati{o}_{PS}$ and zero for $rati{o}_{AOD}$. However, as shown above, their maturation plays an important role for the emergence of spontaneous attractors (see also Supplementary Fig. 4).

## Discussion

We modelled the *in vivo* activity of a developing cortical network during the first postnatal month by combining an extended Wilson-Cowan model with experimentally reported trajectories of neuronal and synaptic parameters. We revealed mechanistically that a particular combination of a transient, hidden unstable state in firing activities and strong synaptic depression enables an immature network to generate large cluster activity while otherwise being mostly silent. We further found that the normal developmental transition from dense to sparse coding is strongly dependent on an elaborate, parallel refinement of absolute synaptic efficacies, both short-term synaptic plasticity (STP) and intrinsic membrane properties, and background activity. Strikingly, in our model, sparsification translates not only to a reduction of postnatal instability of network activity but also to an effective availability of the inhibition-stabilized network (ISN) regime as well as the emergence of spontaneous attractors, providing a novel mechanistic explanation for how the network’s information processing is refined towards eye-opening.

How can immature networks be quiescent for relatively long periods and occasionally generate large cluster activity^3,32^? Surprisingly, we found that while the developing networks (P3 to P20) are operating at their rest state (Fig. 3a), an unstable state is formed in their fast (i.e. firing activity) dynamics (Figs 2e and 3b). This may be a key to this biphasic behavior (Fig. 3c and Supplementary Fig. 2c). In addition, we found that at early stages prior to eye-opening (P3 and P10) immature networks are mono-stable, where the only FP of the network is its stable rest state (Fig. 3a). Accordingly, the underlying mechanism of cluster activity emergence in these networks is in stark contrast to models of cluster activity proposed for adult networks, based on the usual assumption of a bi-stable (or multi-stable) network^18,19^. Our finding about the strong effect of the hidden unstable state (Fig. 3b) during the initial phase of network activity at the rest state (see Figs 2 and 3c) may provide an explanation why neonatal networks, e.g. at P3 and P10, are more susceptible to seizures than mature networks^32,33^. Moreover, the lack of any spontaneous attractor in these networks (Fig. 3a) might also contribute to this susceptibility, since the attractors can, in principle, aid in stabilizing a network’s activity.

Sparsification coincides with the peak of GABAergic and glutamatergic synaptogenesis^11,12^ and overlaps with a developmental reduction in release probabilities, particularly at glutamatergic synapses^10,12^. Using a computational study, we here revealed how these changes are suited to drive the transition from dense to sparse coding during network maturation (e.g. see Figs 3 and 5). Since sensory deprivation affects the total synapse numbers only modestly^34^, our results might also explain previous experimental findings: While sensory experience may have a modulatory effect on sparsification during the first days after eye-opening^3^, this process is largely mediated internally, i.e. independent of sensory inputs^2,3,7^.

How does the sparsification process prepare *in vivo* developing networks for effective sensory processing? While the answer to this question remains poorly understood by experimental studies, our computational study provides two mechanistic insights: Firstly, we found that Non-ISN and instability may play key roles as the dominating operating regimes prior to eye-opening (Fig. 3a), possibly reflecting an immaturity of inhibitory transmission. That is, our modeling results imply that during stimulation (e.g. in response to long-lasting external inputs) the immature networks will operate under a Non-ISN regime, rather than an ISN regime (P3 and P10; Supplementary Fig. 2b), due to the lack of an effective availability of the ISN regime during early development (P3 and P10; Fig. 3a). The Non-ISN regime will enable the immature networks to maintain their stability without requiring inhibitory transmission effects (see Methods). For adult networks, however, some experimental evidence indicates that cortical networks operate as ISNs^25,28^. Strikingly, in the model, during sparsification, the unstable FP-domain is effectively replaced by that of an ISN regime (Fig. 3a and e). This may enable cortical networks to process complex sensory computations^27^ in parallel to the developmental strengthening of sensory inputs.

Secondly, we found that spontaneous attractors start emerging around eye-opening, which, in our model, is mainly due to the combined effect of developmental increase in background activity^2,3,7^ and the developmental decrease in membrane resistance^2,10^. In general, attractors can represent the solution to a specific neural computation^35^. They are also thought to be the substrate of, and used to model, e.g., the working memory^36^, eye position stability^37^, and orientation selectivity^38^. Besides, in our developing model, the attractor emergence phenomenon can also underlie three developmental mechanisms. First, it provides the basis for transitioning between FPs (here, via bi-stable cluster activity at P14 and P20; Fig. 3c) and may be thought of as an early step towards representation of perceptual stimuli. Second, it enables inhibitory transmission to contribute effectively more to network activity, e.g. through providing a sufficiently strong balancing of the intrinsic instability of the E-activity under an ISN regime^26,27^ (P14 and P20; Fig. 3c), or by becoming amenable to terminate activity at the attractor^22^ (Supplementary Fig. 2d). This may be important for a presumably more effective processing of sensory inputs. Third, this phenomenon may also initiate the effective interaction of spontaneous activity with sensory cortical responses, as observed experimentally^17^.

Evidence from *in vitro* studies suggests that GABA depolarizes neonatal neurons, e.g. in rodents during the first postnatal week^20,32^. However, the effect of GABA at the network level is still an open question. While most of the previous *in vitro* studies reported that GABA is excitatory (or both excitatory and inhibitory) at the network level^32^, recent *in vivo* studies harnessing new experimental techniques found that GABA inhibits intact neonatal networks in neocortex and hippocampus^20,29,31^. In particular, GABAergic transmission was shown to limit the spatial extent of cluster activity^20^. In our model, we tested these two hypotheses by blocking the GABAergic receptors in two P3 networks with either excitatory (Supplementary Fig. 6b) or inhibitory GABAergic transmission (Fig. 4b). This simulated manipulation led to an increase in the cluster activity size only when GABAergic transmission was inhibitory at the network level. Therefore, our results support the recent *in vivo* findings^31,33^. Importantly, this finding implies that even when GABA depolarizes immature neurons, GABA still can attenuate the instability effect on network activity, thereby restricting cluster activity size.

To our knowledge, there are only few previous modelling studies covering this developmental period (e.g. see refs^39,40^), where the authors’ main focus was on the potential mechanism of the cluster activity generation under *in vitro* condition. These studies were focused on a single postnatal stage and this stage was mostly restricted to the first postnatal week, like P5. Moreover, these authors probably did not consider some important biophysical considerations which have only recently been revealed by *in vivo* studies: the abolishment of cluster activity following the removal of glutamatergic synapses^20,29^, the existence of a profound inhibitory effect of GABAergic transmission at the network level^20,29^ and its non-excitatory (but depolarizing) effect at the neuron level^20,31^ during the first postnatal week, or that the loss of large cluster activity occurs around eye-opening^2,3^ and not around P7^41^. In contrast, here, (i) we used a unified computational model which takes into account most of these recently reported biophysical considerations (see Supplementary Method and Table 1), (ii) our work shows results based on, and mainly in accordance to the recent *in vivo* studies, where these studies revealed some critical features different from those reported for *in vitro* data, (iii) we studied the whole developmental period, comprising the time course of sparsification, and not only a single postnatal stage, (iv) we not only address the possible underlying mechanism of the postnatal cluster activity generation, but also the developmental changes in the operating regimes and information processing capabilities, as well as the possible refinements in network properties governing the sparsification.

There are advantages and limitations of our modeling approach. Clearly, as compared to a mean-field model as employed here, a spiking network model can in principle provide more detailed results, e.g. one could model the temporal sparseness of spiking activities, or the specific patterns of sensory inputs at the neuron level. However, for this early developmental period, we are not aware of any established neuron model. For such a model, a considerable amount of unspecified neurobiological parameters is required to be measured experimentally first. Instead, using a mean-field model enabled us to forgo such detailed parameterizations by considering only their average characteristics over the network. Although this procedure comes at the price of removing biological details, this enabled us to use an extended Wilson-Cowan-type model^13^; a well-established model and extensively used before to study adult networks behavior^18,42^. This model enabled us to incorporate the available, experimentally reported developmental trajectories of intrinsic neuronal and synaptic parameters (see Supplementary Method and Table 1), including short-term synaptic plasticity. In addition to its biophysical interpretability, this model is in general mathematically tractable which allowed us to derive analytical expressions, e.g. in our stability analyses (see Supplementary Methods). Moreover, this model is readily extendable to incorporate other biophysical mechanisms like spike-frequency adaptation, or to build a spatial graph of multiple homogenous and heterogeneous inter-connected networks to study, e.g. the activity propagation over different brain areas.

In sum, we have shown that by establishing and extending a novel application of an existing computational model to immature networks and integrating recent experimental findings, new mechanistic insights into the development of neural networks can be obtained. We expect that, in the future, this modelling approach can also guide research by providing for concrete predictions that can be tested experimentally.

## Methods

Here, we briefly describe the main components of our model and the analyses. A detailed description can be found in Supplementary Methods.

### STP-RNN model

This model (Fig. 1a) is an extended version of Wilson-Cowan’s recurrent neural network (RNN) mean-field model^43^, for which the short-term plasticity (STP) of synaptic connections were also modeled^13^. The equations governing the model dynamics over time are (dots denote the time derivatives):

$$\begin{array}{rcl}{\tau }_{{\rm{E}}}{\dot{E}}_{{\rm{r}}}(t) & = & -{E}_{{\rm{r}}}(t)+{G}_{{\rm{E}}}({J}_{{\rm{EE}}}\,{u}_{{\rm{EE}}}(t)\,{x}_{{\rm{EE}}}(t)\,{E}_{{\rm{r}}}(t)-{J}_{{\rm{EI}}}\,{u}_{{\rm{EI}}}(t)\,{x}_{{\rm{EI}}}(t)\,{I}_{{\rm{r}}}(t)+{e}_{{\rm{E}}}(t)-{\theta }_{{\rm{E}}})\\ {\tau }_{{\rm{I}}}{\dot{I}}_{{\rm{r}}}(t) & = & -{I}_{{\rm{r}}}(t)+{G}_{{\rm{I}}}({J}_{{\rm{IE}}}\,{u}_{{\rm{IE}}}(t)\,{x}_{{\rm{IE}}}(t)\,{E}_{{\rm{r}}}(t)-{J}_{{\rm{II}}}\,{u}_{{\rm{II}}}(t)\,{x}_{{\rm{II}}}(t)\,{I}_{{\rm{r}}}(t)+{e}_{{\rm{I}}}(t)-{\theta }_{{\rm{I}}})\\ {\dot{x}}_{{\rm{ij}}} & = & {\tau }_{{\rm{r}}{}_{{\rm{ij}}}}^{-1}(1-{x}_{{\rm{ij}}}(t))-{u}_{{\rm{ij}}}(t)\,{x}_{{\rm{ij}}}(t){A}_{{\rm{j}}}(t)\\ {\dot{u}}_{{\rm{ij}}} & = & {\tau }_{{{\rm{f}}}_{{\rm{ij}}}}^{-1}({U}_{{\rm{ij}}}-{u}_{{\rm{ij}}}(t))+{U}_{{\rm{ij}}}(1-{u}_{{\rm{ij}}}(t)){A}_{{\rm{j}}}(t)\end{array}$$

where j and ${\rm{i}}\in \{{\rm{E}},{\rm{I}}\}$, ${A}_{{\rm{j}}}\in \{{E}_{{\rm{r}}}\,,\,{I}_{{\rm{r}}}\}$, j is the index of presynaptic population, ${E}_{{\rm{r}}}$ (=*A*_E_) and ${I}_{{\rm{r}}}$ (=*A*_I_) are the average activity (in hertz) of E- and I-populations which receive, respectively, the external inputs ${e}_{{\rm{E}}}$ and ${e}_{{\rm{I}}}$, e.g., from other brain regions, ${G}_{i}$ is the linear input-output gain above population activity threshold ${\theta }_{i}$ (otherwise ${G}_{i}=0$), and ${x}_{{\rm{ij}}}$ and ${u}_{{\rm{ij}}}$ are the dynamic variables of short-term synaptic depression and facilitation mechanisms. The parameter definitions and values are listed in Table 1. A STP-RNN with synaptic efficacies frozen at the FP is called Frozen STP-RNN (a Static-RNN-type model).

### Phase plane components

*E*_r_-*I*_r_-plane is the 2D phase plane of E- and I-population activity rates (${I}_{{\rm{r}}}$ vs. ${E}_{{\rm{r}}}$), in which the ${E}_{{\rm{r}}}$- and ${I}_{{\rm{r}}}$- nullclines are represented as the set of points for which $d{E}_{{\rm{r}}}/dt=0$ and $d{I}_{{\rm{r}}}/dt=0$, respectively. For a STP-RNN, the depicted quasi ${E}_{{\rm{r}}}$ and ${I}_{{\rm{r}}}$ nullclines in *E*_r_-*I*_r_-plane are based on the reduced STP-RNN (Supplementary equation (7)). Any intersection of these two quasi nullclines (i.e. the FP) can represent the steady state of the whole network (i.e. the 10D STP-RNN).

### Stability of FPs

To determine the stability of any FP of interest we applied linear stability analysis to our 10D STP-RNNs (or 2D Frozen STP-RNNs): We investigated whether all eigenvalues of the network’s system of equations linearized around the FP (using the Jacobian matrix) have strictly negative real parts (if so, the FP is stable), or whether at least one eigenvalue with positive real part exists (if so, the FP is unstable). This can be re-stated as: A FP is stable if following a small perturbation from that FP (at which the network was before perturbation), the network dynamics converge back to it. Conversely, if the dynamics move away from the FP or dies out, that FP is unstable.

### Operating regimes and FP-domains

The stable operating regimes of a RNN at a FP can be classified as an inhibition-stabilized network (ISN) vs. a Non-ISN^25,26^. To discriminate between these two regimes three criteria were defined: (**A**) Excitatory instability: For the inhibitory activity rate fixed at the FP, the recurrent excitation is strong enough to render the E-population intrinsically unstable. (**B**) Excitatory stability: In contrast to (**A**), the E-population is stable *per se*, i.e. even with a feedback inhibition fixed at its level at the FP. (**C**) Overall stability: The dynamic feedback inhibition to the E-population is strong enough to stabilize the whole network activity. At a FP, a network operating under the (**A**) and (**C**) criteria is an ISN, while a network operating under the (**B**) and (**C**) criteria is a Non-ISN. A network, which is neither ISN nor Non-ISN at the FP, operates under an unstable regime.

We partition the *E*_r_-*I*_r_-plane into different domains of operating regimes (FP-domains). Each FP-domain contains all potential steady states (i.e. FPs) at which the network could operate under the corresponding regime. The area of each FP-domain is computed numerically as *AOD* (area of domain). Note that the borders between the FP-domains (Fig. 3a) were determined by using numerical simulations, when we plotted the FP-domains of operating regimes based on their stability criteria (**A**-**C**) obtained analytically in Supplementary Methods (see “Characterization of operating regimes” section in Supplementary Methods).

### Cluster activity size and duration

In a RNN, the cluster activity (network spike; PS_net_) usually involves the population spikes^44,45^ in both E (PS_E_) and I (PS_I_) populations. To approximate cluster activity size, i.e. the total number of neurons synchronized during a cluster activity, we calculate $P{S}_{{\rm{net}}}^{{\rm{amp}}}=\omega \times ({A}_{\,{\rm{sum}}}^{{\rm{amp}}}-{A}_{\,{\rm{sum}}}^{{\rm{0}}})$ (a dimensionless parameter), where *A*_sum_ = *E*_r_ + *I*_r_ is the sum of the activity of E- and I-populations (in hertz), ${A}_{\,{\rm{sum}}}^{{\rm{amp}}}$ and ${A}_{\,{\rm{sum}}}^{{\rm{0}}}$ are the maximal activity during the cluster activity and the preceding activity, respectively, and $\omega$ is a scaling factor (in units of [Hz^−1^]) to convert the activity rate to an approximate number of activated neurons during the cluster activity. For simplicity, we set $\omega =1$, since its veridical value is not defined for the mean-field model. We measure the cluster activity duration as its termination time minus its onset time.

### Computation of ${\boldsymbol{rati}}{{\boldsymbol{o}}}_{{\boldsymbol{PS}}}$ and ${\boldsymbol{rati}}{{\boldsymbol{o}}}_{{\boldsymbol{AOD}}}$

To investigate the impact of specific maturational processes on sparsification (as used in Fig. 5), we first substituted single parameters or a small combination of parameters at P10 by their values at P20 (Table 1), followed by the computation of the $rati{o}_{\gamma }=100\times \varpi \,({\gamma }_{{\rm{P10}}}^{{\rm{res}}}-{\gamma }_{{\rm{P}}10})/({\gamma }_{{\rm{P}}20}-{\gamma }_{{\rm{P}}10})$, where for $rati{o}_{PS}$ we set $\gamma =P{S}_{{\rm{net}}}^{{\rm{amp}}}$ with $\varpi =-1$, and for $rati{o}_{AOD}$ we set *γ* = *AOD*_ISN/Unstable_ with $\varpi =+1$. ${\gamma }_{{\rm{P10}}}^{{\rm{res}}}$ is the value of *γ* measured after the parameter(s) value(s) substitution.

### Data availability

Custom Matlab and Mathematica codes for our model are available upon request from the corresponding author.

## Electronic supplementary material

Supplementary Information
