# Supplementary material for: Developmental Emergence of Sparse Coding: A Dynamic Systems Approach
Source: Sci Rep. 2017 Oct 12;7:13015. doi: 10.1038/s41598-017-13468-z (PMC5638906; doi:10.1038/s41598-017-13468-z)
Supplement: Supplementary file 1 — Supplementary Information [file 41598_2017_13468_MOESM1_ESM.pdf]

Developmental Emergence of Sparse Coding: A Dynamic Systems Approach

Vahid Rahmati, Knut Kirmse, Knut Holthoff, Lars Schwabe, Stefan J. Kiebel

Supplementary Methods

Overview

Our approach to study the developmental changes in the dynamical behavior of immature neural networks is based on four steps. First, we use a mean-field network model that describes the neuronal population dynamics using ordinary differential equations (ODEs). This model is of the Wilson-Cowan-type [1,2] and is presented here without further derivation where biophysical interpretations are available in the literature; e.g. see refs [3,4]. Second, we extend the network model to include short-term synaptic plasticity (STP) mechanisms, namely short-term synaptic depression (STD) and facilitation (STF) [5,6]. Third, we describe the phase plane analysis and computation of nullclines. Fourth, we define cortical operating regimes in terms of steady state properties from the linear stability analysis. Finally, we outline how we chose and set parameter values, for postnatal developing networks.

Model description

The schematic diagram of our two-population recurrent neural network (RNN) model is shown in Fig. 1a. The network consists of two spatially localized, homogeneous excitatory (E) and inhibitory (I) populations with reciprocal connections to each other and self-connections. At any given time, each of the E- and I-populations receives an external input e_{E} and e_{I}, respectively, from other brain regions. These inputs represent e.g. the feedforward inputs to network [7,8]. For ongoing spontaneous activity, we set $e_{\mathrm{E}} = e_{\mathrm{I}} = 0$ [2]. The network dynamics are described by the mean-field equations [1,2]:

$$\begin{aligned} \tau_{\mathrm{E}} \frac{d}{dt} E_{\mathrm{r}} &= -E_{\mathrm{r}} + f_{\mathrm{E}}\left(E_{\mathrm{r}}, I_{\mathrm{r}}, e_{\mathrm{E}}\right) = -E_{\mathrm{r}} + f_{\mathrm{E}}\left(h_{\mathrm{E}}\right) \\ \tau_{\mathrm{I}} \frac{d}{dt} I_{\mathrm{r}} &= -I_{\mathrm{r}} + f_{\mathrm{I}}\left(E_{\mathrm{r}}, I_{\mathrm{r}}, e_{\mathrm{I}}\right) = -I_{\mathrm{r}} + f_{\mathrm{I}}\left(h_{\mathrm{I}}\right) \end{aligned} \quad (1)$$

where E_{r} and I_{r} are the average activity (in hertz) of the E- and I-populations, and τ_{E} and τ_{I} are the time constants of these populations, which are here considered as an approximation of the decay time constants of E (i.e. glutamatergic) and I (i.e. GABAergic) postsynaptic responses; e.g. see refs [4,8]. $f_{\mathrm{E}}\left(h_{\mathrm{E}}\right)$ and $f_{\mathrm{I}}\left(h_{\mathrm{I}}\right)$ are the so-called response functions, which model the transformation from summed inputs, h_{ζ}, to an activity output (in hertz) defined as [5,9]:

$$f_{\zeta}\left(h_{\zeta}\right) = \begin{cases} 0 & \text{for } h_{\zeta} \le \theta_{\zeta} \\ G_{\zeta}\left(h_{\zeta} - \theta_{\zeta}\right) & \text{for } \theta_{\zeta} < h_{\zeta} \end{cases} \quad (2)$$

for $\zeta \in \{\mathrm{E}, \mathrm{I}\}$, where θ_{ζ} are the population activity thresholds [2,5], and G_{ζ} are the linear input-output gains above these thresholds. For the analyses below, we first investigate the networks in the supra-threshold mode (similarly to [1,8]), i.e. when the summed inputs to both populations are larger than their corresponding θ_{ζ}. We then readily generalize our analyses to the three other cases, where either E- or I-

population or both of them are in a sub-threshold mode (see the "Sub-threshold networks" section below). Obviously, for a subthreshold mode the corresponding G_ζ can be considered to be zero.

In the following, we use the mean-field model (equation (1)) to first formulate the dynamics of RNNs with time-independent synaptic efficacies (Static-RNN), and with dynamic efficacies (STP-RNN). To do this, we will extend equation (1) to include STP dynamics.

Static-RNN

In a Static-RNN, the synaptic connections have efficacies, which are constant over time. The dynamics of this network are given by (e.g. see refs [1,8]):

$$\frac{d}{dt}\begin{pmatrix}\tau_{\mathrm{E}} E_{\mathrm{r}} \\ \tau_{\mathrm{I}} I_{\mathrm{r}}\end{pmatrix} = -\begin{pmatrix}E_{\mathrm{r}} \\ I_{\mathrm{r}}\end{pmatrix} + \begin{pmatrix}G_{\mathrm{E}} J_{\mathrm{EE}} & -G_{\mathrm{E}} J_{\mathrm{EI}} \\ G_{\mathrm{I}} J_{\mathrm{IE}} & -G_{\mathrm{I}} J_{\mathrm{II}}\end{pmatrix}\begin{pmatrix}E_{\mathrm{r}} \\ I_{\mathrm{r}}\end{pmatrix} - \begin{pmatrix}G_{\mathrm{E}}\theta_{\mathrm{E}} \\ G_{\mathrm{I}}\theta_{\mathrm{I}}\end{pmatrix} + \begin{pmatrix}G_{\mathrm{E}} e_{\mathrm{E}} \\ G_{\mathrm{I}} e_{\mathrm{I}}\end{pmatrix} \quad (3)$$

where J_{ij} are the average maximum absolute efficacies of synaptic recurrent ($\mathrm{i} = \mathrm{j}$) and feedback connections ($\mathrm{i} \neq \mathrm{j}$); from presynaptic population j to postsynaptic population i, where j and $\mathrm{i} \in \{\mathrm{E}, \mathrm{I}\}$. For example, J_{EE} denotes the average number of recurrent excitatory contacts per each postsynaptic neuron in the E-population, times the average amplitude of unitary maximal postsynaptic response on the neuron [1,5].

STP-RNN

STP [6,10] renders the synaptic efficacies dynamic over time, as a function of the recent history of presynaptic activity. The main underlying biological mechanisms of STP are [6]: i) vesicle depletion and ii) accumulation of calcium at the presynaptic axon terminals, during the neurotransmission. These two have an opposite impact on synaptic efficacy: while the depletion decreases the synaptic efficacy (short-term depression; STD), the accumulation increases it (short-term facilitation; STF). For including these mechanisms in the mean-field equations (equation (1)), we follow the Tsodyks-Markram approach [5]. In their model, STD and STF mechanisms are governed by two STP variables per synaptic connection: x_{ij} (average fraction of available synaptic resources) and u_{ij} (average utilization parameter), respectively. For instance, in the case of a spike arrival at a single axon terminal, u_{ij} is facilitated due to spike-evoked calcium influx to the terminal, and then decays back to its basal value U_{ij} (analogous to the release probability) with the time constant $\tau_{\mathrm{f_{ij}}}$, while x_{ij} is "consumed" with the spike and then recovers to its baseline value of 1 with time constant $\tau_{\mathrm{r_{ij}}}$. Accordingly, the mean-field dynamics of a STP-RNN are described by [5]:

$$\frac{d}{dt}\begin{pmatrix}\tau_{\mathrm{E}}E_{\mathrm{r}}\\ \tau_{\mathrm{I}}I_{\mathrm{r}}\end{pmatrix}=-\begin{pmatrix}E_{\mathrm{r}}\\ I_{\mathrm{r}}\end{pmatrix}+\begin{pmatrix}G_{\mathrm{E}}J_{\mathrm{EE}}u_{\mathrm{EE}}x_{\mathrm{EE}} & -G_{\mathrm{E}}J_{\mathrm{EI}}u_{\mathrm{EI}}x_{\mathrm{EI}}\\ G_{\mathrm{I}}J_{\mathrm{IE}}u_{\mathrm{IE}}x_{\mathrm{IE}} & -G_{\mathrm{I}}J_{\mathrm{II}}u_{\mathrm{II}}x_{\mathrm{II}}\end{pmatrix}\begin{pmatrix}E_{\mathrm{r}}\\ I_{\mathrm{r}}\end{pmatrix}-\begin{pmatrix}G_{\mathrm{E}}\theta_{\mathrm{E}}\\ G_{\mathrm{I}}\theta_{\mathrm{I}}\end{pmatrix}+\begin{pmatrix}G_{\mathrm{E}}e_{\mathrm{E}}\\ G_{\mathrm{I}}e_{\mathrm{I}}\end{pmatrix}$$
$$\frac{d}{dt}x_{\mathrm{ij}}=\frac{1-x_{\mathrm{ij}}}{\tau_{r_{\mathrm{ij}}}}-u_{\mathrm{ij}}x_{\mathrm{ij}}A_{\mathrm{j}} \quad (4)$$
$$\frac{d}{dt}u_{\mathrm{ij}}=\frac{U_{\mathrm{ij}}-u_{\mathrm{ij}}}{\tau_{\mathrm{f_{ij}}}}+U_{\mathrm{ij}}\left(1-u_{\mathrm{ij}}\right)A_{\mathrm{j}}$$

where $A_{\mathrm{j}}\in\{E_{\mathrm{r}},I_{\mathrm{r}}\}$, and $\mathrm{j}\in\{\mathrm{E},\mathrm{I}\}$ is the index of the presynaptic population ($A_{\mathrm{E}}=E_{\mathrm{r}}$ and $A_{\mathrm{I}}=I_{\mathrm{r}}$). We model each postnatal developing neural network as a STP-RNN.

Phase plane and nullclines

To characterize the operating regimes of the Static-RNN and STP-RNN, we use phase planes and linear stability analyses. In the following we start by formulating the corresponding stationary components and properties, including the steady state values and the slopes of nullclines.

Conditions for steady states

For each network, the steady state values of its dynamics can be obtained by setting its ODEs equal to zero. Such states may be achieved e.g. in response to sustained input. We have listed the steady state values and the resulting steady state connectivity matrices in Supplementary Table 1. In contrast to the Static-RNN, the STP variables of STP-RNN may converge to steady states as:

$$u_{\mathrm{ij}}^{\mathrm{ss}}x_{\mathrm{ij}}^{\mathrm{ss}}=\frac{A_{\mathrm{j}}\tau_{\mathrm{f_{ij}}}+1}{A_{\mathrm{j}}^{2}\tau_{\mathrm{f_{ij}}}\tau_{\mathrm{r_{ij}}}+A_{\mathrm{j}}\left(\tau_{\mathrm{f_{ij}}}+\tau_{\mathrm{r_{ij}}}\right)+\frac{1}{U_{\mathrm{ij}}}} \quad (5)$$

which implies that different sustained levels of the presynaptic activity ($A_{\mathrm{j}}\in\{E_{\mathrm{r}},I_{\mathrm{r}}\}$) can lead to different steady state efficacies (see $\mathbf{J}_{\mathrm{ss}}^{\mathrm{STP}}$ in Supplementary Table 1).

Computation of 2D phase planes

The rationale of our approach is that the operating regimes we are interested in (such as an inhibition-stabilized network (ISN) vs. Non-ISN; see below) can be defined at the intersection of the excitatory and inhibitory nullclines, namely at the fixed point (FP) [1,8]. For Static-RNN the nullclines of the E- and I-populations (E_{r}- nullcline and I_{r}- nullcline) in a 2D phase plane (E_{r}-I_{r}-plane) are computed as:

$$\Theta_{\mathrm{E}}^{\mathrm{STC}}\left(E_{\mathrm{r}},I_{\mathrm{r}}\right)=-E_{\mathrm{r}}+G_{\mathrm{E}}\left(J_{\mathrm{EE}}E_{\mathrm{r}}-J_{\mathrm{EI}}I_{\mathrm{r}}-\theta_{\mathrm{E}}+e_{\mathrm{E}}\right)=0$$
$$\Theta_{\mathrm{I}}^{\mathrm{STC}}\left(E_{\mathrm{r}},I_{\mathrm{r}}\right)=-I_{\mathrm{r}}+G_{\mathrm{I}}\left(J_{\mathrm{IE}}E_{\mathrm{r}}-J_{\mathrm{II}}I_{\mathrm{r}}-\theta_{\mathrm{I}}+e_{\mathrm{I}}\right)=0 \quad (6)$$

where $\Theta_{\mathrm{E}}^{\mathrm{STC}}$ and $\Theta_{\mathrm{I}}^{\mathrm{STC}}$ are the right-hand sides of the ODEs in equation (3). However, this two-dimensional E_{r}-I_{r}-plane analysis [1,2] is not directly applicable to STP-RNNs, since it is a 10D system (8 STP and 2 activity variables; see equation (4)) with ten 10D nullclines. Instead, for this network we compute

the quasi E_r- and I_r- nullclines in the E_r-I_r-plane- like (or simply E_r-I_r-plane, for brevity) sketches. To do this, we assume that at each FP, the synaptic efficacies of the STP-RNN are at their steady state values (see Supplementary Table 1), where these steady states are in turn a function of the activity variables determined at that point (see equation (5), and $\mathbf{J}_\mathrm{ss}^\mathrm{STP}$ in Supplementary Table 1). Accordingly, by substituting the STP variables in the activity ODEs (equation (4)) with their steady state values (see equation (5)), the network dimensionality is reduced from ten to two:

$$\frac{d}{dt}\begin{pmatrix}\tau_\mathrm{E}E_\mathrm{r}\\ \tau_\mathrm{I}I_\mathrm{r}\end{pmatrix} = -\begin{pmatrix}E_\mathrm{r}\\ I_\mathrm{r}\end{pmatrix} + \begin{pmatrix}G_\mathrm{E}\,J_\mathrm{EE}\,u_\mathrm{EE}^\mathrm{ss}\,x_\mathrm{EE}^\mathrm{ss} & -G_\mathrm{E}\,J_\mathrm{EI}\,u_\mathrm{EI}^\mathrm{ss}\,x_\mathrm{EI}^\mathrm{ss}\\ G_\mathrm{I}\,J_\mathrm{IE}\,u_\mathrm{IE}^\mathrm{ss}\,x_\mathrm{IE}^\mathrm{ss} & -G_\mathrm{I}\,J_\mathrm{II}\,u_\mathrm{II}^\mathrm{ss}\,x_\mathrm{II}^\mathrm{ss}\end{pmatrix}\begin{pmatrix}E_\mathrm{r}\\ I_\mathrm{r}\end{pmatrix} - \begin{pmatrix}G_\mathrm{E}\theta_\mathrm{E}\\ G_\mathrm{I}\theta_\mathrm{I}\end{pmatrix} + \begin{pmatrix}G_\mathrm{E}e_\mathrm{E}\\ G_\mathrm{I}e_\mathrm{I}\end{pmatrix} \quad (7)$$

and thus, we can compute the (2D) quasi E_r- and I_r- nullclines as:

$$\begin{aligned}\tilde{\Theta}_\mathrm{E}^\mathrm{STP}\left(E_\mathrm{r},I_\mathrm{r}\right) &= -E_\mathrm{r} + G_\mathrm{E}\left(J_\mathrm{EE}\,u_\mathrm{EE}^\mathrm{ss}\,x_\mathrm{EE}^\mathrm{ss}\,E_\mathrm{r} - J_\mathrm{EI}\,u_\mathrm{EI}^\mathrm{ss}\,x_\mathrm{EI}^\mathrm{ss}\,I_\mathrm{r} - \theta_\mathrm{E} + e_\mathrm{E}\right) = 0\\ \tilde{\Theta}_\mathrm{I}^\mathrm{STP}\left(E_\mathrm{r},I_\mathrm{r}\right) &= -I_\mathrm{r} + G_\mathrm{I}\left(J_\mathrm{IE}\,u_\mathrm{IE}^\mathrm{ss}\,x_\mathrm{IE}^\mathrm{ss}\,E_\mathrm{r} - J_\mathrm{II}\,u_\mathrm{II}^\mathrm{ss}\,x_\mathrm{II}^\mathrm{ss}\,I_\mathrm{r} - \theta_\mathrm{I} + e_\mathrm{I}\right) = 0\end{aligned} \quad (8)$$

where $\tilde{\Theta}_\mathrm{E}^\mathrm{STP}$ and $\tilde{\Theta}_\mathrm{I}^\mathrm{STP}$ are the right-hand sides of the ODEs in equation (7). Substituting $u_\mathrm{ij}^\mathrm{ss}x_\mathrm{ij}^\mathrm{ss}$ with their steady state expressions (as in equation (5)) makes the quasi E_r- and I_r- nullclines just dependent on the activity variables (but nonlinearly, as opposed to the case of Static-RNN). Most importantly, this implies that the networks operating at different FPs have, in general, STP variables (thus, synaptic efficacies) with different steady state values (see $\mathbf{J}_\mathrm{ss}^\mathrm{STP}$ in Supplementary Table 1), depending on the underlying activity rates. Moreover, note that the intersection of the quasi E_r- and I_r- nullclines in the non-negative ranges of E_r-I_r-plane will capture the FPs of the full system (i.e. the 10D STP-RNN, equation (4)).

Frozen STP-RNN

Before proceeding with our analyses, we first introduce the Frozen STP-RNN which allowed us to characterize the dynamics of the STP-RNN using analytical expressions (see the "Characterization of operating regimes" section, below). To investigate the effect of STP on the fast network dynamics we can freeze (i.e., fix) the synaptic variables at their steady state values at the FP of interest ("frozen weight"). This approximation may be justified as the initial phase of the network response mainly depends on the activity variables, which evolve much faster than STP variables, see also ref. [9]. Effectively, the freezing converts the STP-RNN to a Static-RNN model with the connectivity matrix determined at that FP. Accordingly, the dynamics of a Frozen STP-RNN (2D) can be readily adapted from equation (3) (see also Supplementary Table 1, for steady states values):

$$\frac{d}{dt}\begin{pmatrix}\tau_\mathrm{E}E_\mathrm{r}\\ \tau_\mathrm{I}I_\mathrm{r}\end{pmatrix} = -\begin{pmatrix}E_\mathrm{r}\\ I_\mathrm{r}\end{pmatrix} + \begin{pmatrix}G_\mathrm{E}J_\mathrm{EE}^\mathrm{FP} & -G_\mathrm{E}J_\mathrm{EI}^\mathrm{FP}\\ G_\mathrm{I}J_\mathrm{IE}^\mathrm{FP} & -G_\mathrm{I}J_\mathrm{II}^\mathrm{FP}\end{pmatrix}\begin{pmatrix}E_\mathrm{r}\\ I_\mathrm{r}\end{pmatrix} - \begin{pmatrix}G_\mathrm{E}\theta_\mathrm{E}\\ G_\mathrm{I}\theta_\mathrm{I}\end{pmatrix} + \begin{pmatrix}G_\mathrm{E}e_\mathrm{E}\\ G_\mathrm{I}e_\mathrm{I}\end{pmatrix} \quad (9)$$

where $J_\mathrm{ij}^\mathrm{FP} = J_\mathrm{ij}u_\mathrm{ij}^\mathrm{FP}x_\mathrm{ij}^\mathrm{FP}$, and $u_\mathrm{ij}^\mathrm{FP}x_\mathrm{ij}^\mathrm{FP}$ are the values of $u_\mathrm{ij}^\mathrm{ss}x_\mathrm{ij}^\mathrm{ss}$ (see equation (5)) at the FP of interest. Similarly, its E_r- and I_r- nullclines are obtained as:

$$\Theta_{\mathrm{E}}^{\mathrm{STC}}\left(E_{\mathrm{r}},I_{\mathrm{r}}\right)=-E_{\mathrm{r}}+G_{\mathrm{E}}\left(J_{\mathrm{EE}}^{\mathrm{FP}}E_{\mathrm{r}}-J_{\mathrm{EI}}^{\mathrm{FP}}I_{\mathrm{r}}-\theta_{\mathrm{E}}+e_{\mathrm{E}}\right)=0$$
$$\Theta_{\mathrm{I}}^{\mathrm{STC}}\left(E_{\mathrm{r}},I_{\mathrm{r}}\right)=-I_{\mathrm{r}}+G_{\mathrm{I}}\left(J_{\mathrm{IE}}^{\mathrm{FP}}E_{\mathrm{r}}-J_{\mathrm{II}}^{\mathrm{FP}}I_{\mathrm{r}}-\theta_{\mathrm{I}}+e_{\mathrm{I}}\right)=0 \quad (10)$$

Importantly, note that the Frozen STP-RNN not only can be used to reliably determine the operating regime of the STP-RNN at FP (see “Characterization of operating regimes” section), but also enables one to compare the transient behavior of the two network types (STP-RNN vs. Static-RNN) in response to e.g. a perturbation, at the FP (see Results).

Properties of nullclines

To characterize the operating regimes we also consider the slopes of the activity nullclines in the E_{r}-I_{r}-plane (I_{r} vs. E_{r}). In our preliminary analysis, we found that the operating regime of a developing network (i.e. a STP-RNN, equation (4)) at the FP can be reliably determined based on its corresponding Frozen STP-RNN (equation (9)), rather than the reduced system defined in equation (7) (and its quasi nullclines). Therefore, for our slope analysis we only need to consider the Frozen STP-RNN. However, as mentioned above (see the text immediately following equation (8)), the reduced system in equation (7) can capture the FPs of the full STP-RNN (equation (4)). Accordingly, to track how the location of the FPs may change in the E_{r}-I_{r}-plane (non-negative ranges), as well as for the sake of generality, below we first develop a generic framework for formulating such slopes for the reduced STP-RNN (equation (7)), and then apply it to the Frozen STPP-RNN. In particular, as we found in our simulations that the quasi E_{r}- nullcline has in general a non-monotonic shape (see Results), we will also test whether the quasi nullclines or the nullclines of the Frozen STP-RNN exhibit any extremum in the E_{r}-I_{r}-plane.

Slopes of STP-RNN’s quasi nullclines

We first re-write the expressions of quasi nullclines in equation (8) as:

$$\tilde{\Theta}_{\mathrm{E}}^{\mathrm{STP}}\left(E_{\mathrm{r}},I_{\mathrm{r}}\right)=E_{\mathrm{r}}\psi_{\mathrm{EE}}\left(E_{\mathrm{r}}\right)-I_{\mathrm{r}}\psi_{\mathrm{EI}}\left(I_{\mathrm{r}}\right)+G_{\mathrm{E}}\left(e_{\mathrm{E}}-\theta_{\mathrm{E}}\right)=0$$
$$\tilde{\Theta}_{\mathrm{I}}^{\mathrm{STP}}\left(E_{\mathrm{r}},I_{\mathrm{r}}\right)=-I_{\mathrm{r}}\psi_{\mathrm{II}}\left(I_{\mathrm{r}}\right)+E_{\mathrm{r}}\psi_{\mathrm{IE}}\left(E_{\mathrm{r}}\right)+G_{\mathrm{I}}\left(e_{\mathrm{I}}-\theta_{\mathrm{I}}\right)=0 \quad (11)$$

where $\psi_{\mathrm{EE}}\left(E_{\mathrm{r}}\right)=G_{\mathrm{E}}\left(J_{\mathrm{EE}}u_{\mathrm{EE}}^{\mathrm{ss}}x_{\mathrm{EE}}^{\mathrm{ss}}\right)-1$, $\psi_{\mathrm{EI}}\left(I_{\mathrm{r}}\right)=G_{\mathrm{E}}J_{\mathrm{EI}}u_{\mathrm{EI}}^{\mathrm{ss}}x_{\mathrm{EI}}^{\mathrm{ss}}$, $\psi_{\mathrm{II}}\left(I_{\mathrm{r}}\right)=G_{\mathrm{I}}J_{\mathrm{II}}u_{\mathrm{II}}^{\mathrm{ss}}x_{\mathrm{II}}^{\mathrm{ss}}+1$ and $\psi_{\mathrm{IE}}\left(E_{\mathrm{r}}\right)=G_{\mathrm{I}}J_{\mathrm{IE}}u_{\mathrm{IE}}^{\mathrm{ss}}x_{\mathrm{IE}}^{\mathrm{ss}}$ are implicit functions of activity variables. Hence, by using implicit differentiations we can compute:

$$\frac{d\tilde{\Theta}_{\mathrm{E}}^{\mathrm{STP}}\left(E_{\mathrm{r}},I_{\mathrm{r}}\right)}{dE_{\mathrm{r}}}=\psi_{\mathrm{EE}}\left(E_{\mathrm{r}}\right)+E_{\mathrm{r}}\frac{d\psi_{\mathrm{EE}}\left(E_{\mathrm{r}}\right)}{dE_{\mathrm{r}}}-\frac{dI_{\mathrm{r}}}{dE_{\mathrm{r}}}\psi_{\mathrm{EI}}\left(I_{\mathrm{r}}\right)-I_{\mathrm{r}}\frac{d\psi_{\mathrm{EI}}\left(I_{\mathrm{r}}\right)}{dE_{\mathrm{r}}}=0$$
$$\frac{d\tilde{\Theta}_{\mathrm{I}}^{\mathrm{STP}}\left(E_{\mathrm{r}},I_{\mathrm{r}}\right)}{dE_{\mathrm{r}}}=-\frac{dI_{\mathrm{r}}}{dE_{\mathrm{r}}}\psi_{\mathrm{II}}\left(I_{\mathrm{r}}\right)-I_{\mathrm{r}}\frac{d\psi_{\mathrm{II}}\left(I_{\mathrm{r}}\right)}{dE_{\mathrm{r}}}+\psi_{\mathrm{IE}}\left(E_{\mathrm{r}}\right)+E_{\mathrm{r}}\frac{d\psi_{\mathrm{IE}}\left(E_{\mathrm{r}}\right)}{dE_{\mathrm{r}}}=0 \quad (12)$$

where $d\psi_{\mathrm{EI}}\left(I_{\mathrm{r}}\right)/dE_{\mathrm{r}}=\left(d\psi_{\mathrm{EI}}\left(I_{\mathrm{r}}\right)/dI_{\mathrm{r}}\right)\times\left(dI_{\mathrm{r}}/dE_{\mathrm{r}}\right)$ and $d\psi_{\mathrm{II}}\left(I_{\mathrm{r}}\right)/dE_{\mathrm{r}}=\left(d\psi_{\mathrm{II}}\left(I_{\mathrm{r}}\right)/dI_{\mathrm{r}}\right)\times\left(dI_{\mathrm{r}}/dE_{\mathrm{r}}\right)$. By substituting these terms into equation (12), the general expression for the slopes of quasi E_{r}- and I_{r}-nullclines ($slope_{\mathrm{E}}^{\text{STP-like}}$ and $slope_{\mathrm{I}}^{\text{STP-like}}$) are obtained analytically as:

$$
\begin{aligned}
slope_{\mathrm{E}}^{\text{STP-like}} &= \frac{dI_r}{dE_{\mathrm{r}}}=\frac{\psi_{\mathrm{EE}}\left(E_{\mathrm{r}}\right)+E_{\mathrm{r}}\left(d\psi_{\mathrm{EE}}\left(E_{\mathrm{r}}\right)/dE_{\mathrm{r}}\right)}{\psi_{\mathrm{EI}}\left(I_{\mathrm{r}}\right)+I_{\mathrm{r}}\left(d\psi_{\mathrm{EI}}\left(I_{\mathrm{r}}\right)/dI_{\mathrm{r}}\right)} \\
slope_{\mathrm{I}}^{\text{STP-like}} &= \frac{dI_{\mathrm{r}}}{dE_{\mathrm{r}}}=\frac{\psi_{\mathrm{IE}}\left(E_{\mathrm{r}}\right)+E_{\mathrm{r}}\left(d\psi_{\mathrm{IE}}\left(E_{\mathrm{r}}\right)/dE_{\mathrm{r}}\right)}{\psi_{\mathrm{II}}\left(I_{\mathrm{r}}\right)+I_{\mathrm{r}}\left(d\psi_{\mathrm{II}}\left(I_{\mathrm{r}}\right)/dI_{\mathrm{r}}\right)}
\end{aligned}
\quad (13)
$$

Note that these slopes do not depend on activity-independent parameters like the inputs or the population activity thresholds (e.g. see equation (4)).

We now investigate the possibility of a sign-switch in these slopes; each sign-switch implies the existence of one extremum leading to a non-monotonic form of the related nullcline in the E_{r}-I_{r}-plane. To do this, we start by checking the following necessary (but not sufficient) condition: The slope is zero at an extremum. Accordingly, we set each of the computed slopes (equation (13)) equal to zero, and search for the activity rates at which extrema may occur. For a quasi I_{r}-nullcline to have a sign-switch at least one of the following two conditions needs to be fulfilled:

$$\left(U_{\mathrm{IE}}\tau_{\mathrm{f_{IE}}}^{2}\right)E_{\mathrm{r}}^{2}+2\tau_{\mathrm{f_{IE}}}E_{\mathrm{r}}+1=0 \quad (\mathrm{I})$$

$$G_I J_{IE} U_{IE}\left(\left(\tau_{\mathrm{f_{II}}}\tau_{\mathrm{r_{II}}}\right)I_{\mathrm{r}}^{2}+\left(\tau_{\mathrm{f_{II}}}+\tau_{\mathrm{r_{II}}}\right)I_r+U_{\mathrm{II}}^{-1}\right)^{2}=0 \quad (\mathrm{II})$$

The roots of the first polynomial (condition (I)) are $E_{\mathrm{r}}^{1,2}=\left(-1\pm\sqrt{1-U_{\mathrm{IE}}}\right)/U_{\mathrm{IE}}\tau_{\mathrm{f_{IE}}}$. As the roots, we only accept the real positive activity rates; see also ref. [9]. In our modelling, for all connections we have $0\le U_{\mathrm{ij}}\le 1$ (analogous to release probability). Therefore, the constraint on having a real root is always fulfilled, since $1-U_{\mathrm{IE}}\ge 0$. However, $-1\pm\sqrt{1-U_{\mathrm{IE}}}\le 0$. Hence, the first polynomial does not have any admissible (i.e. real positive) root, meaning that the slope-sign of the quasi I_{r}-nullcline cannot change due to condition (I). Alternatively, this could also be found by using Vieta's formulas, which relates the coefficients of a polynomial to the sum and product of its roots. For our quadratic polynomial in condition (I), applying this formula results in:

$$E_{\mathrm{r}}^{1}+E_{\mathrm{r}}^{2}=\frac{-2}{U_{\mathrm{IE}}\tau_{\mathrm{f_{IE}}}}<0 \quad \& \quad E_{\mathrm{r}}^{1}E_{\mathrm{r}}^{2}=\frac{1}{U_{\mathrm{IE}}\tau_{\mathrm{f_{IE}}}^{2}}>0 \quad (14)$$

which imply that both roots have negative real parts, and thus are not admissible. Similarly, by applying this theorem to the repeated roots of the quadratic polynomial in condition (II), we obtain:

$$I_{\mathrm{r}}^{1}+I_{\mathrm{r}}^{2}=-\frac{\tau_{\mathrm{f_{II}}}+\tau_{\mathrm{r_{II}}}}{\tau_{\mathrm{f_{II}}}\tau_{\mathrm{r_{II}}}}<0 \quad \& \quad I_{\mathrm{r}}^{1}I_{\mathrm{r}}^{2}=\frac{1}{U_{\mathrm{II}}\tau_{\mathrm{f_{II}}}\tau_{\mathrm{r_{II}}}}>0 \quad (15)$$

which show that these roots are also negative (although not shown, the same results about condition (II) could also be derived using the direct way as used additionally for condition (I)). Overall, we can conclude that the $slope_{\mathrm{I}}^{\text{STP-like}}$ does not include any sign-switch. Besides, since the polynomials in conditions (I) and (II), whose multiplication determines the numerator, and the denominator (not shown) of $slope_{\mathrm{I}}^{\text{STP-like}}$ contain only positive quantities, the quasi I_{r}-nullcline has always a monotonically increasing slope in the E_{r}-I_{r}-plane, for positive activity rates.

For the quasi E_{r}-nullcline, setting $slope_{\mathrm{E}}^{\text{STP-like}}=0$ yields the following conditions:

$$\left(\left(\tau_{\mathrm{f_{EI}}}\tau_{\mathrm{r_{EI}}}\right)I_{\mathrm{r}}^{2}+\left(\tau_{\mathrm{f_{EI}}}+\tau_{\mathrm{r_{EI}}}\right)I_{\mathrm{r}}+U_{\mathrm{EI}}^{-1}\right)^{2}=0 \quad (\mathrm{III})$$

$$-\left(\left(\tau_{\mathrm{f_{EE}}}\tau_{\mathrm{r_{EE}}}\right)E_{\mathrm{r}}^{2}+\left(\tau_{\mathrm{f_{EE}}}+\tau_{\mathrm{r_{EE}}}\right)E_{\mathrm{r}}+U_{\mathrm{EE}}^{-1}\right)^{2}+G_{\mathrm{E}}J_{\mathrm{EE}}U_{\mathrm{EE}}^{-1}\left(1+\tau_{\mathrm{f_{EE}}}E_{\mathrm{r}}\left(\tau_{\mathrm{f_{EE}}}U_{\mathrm{EE}}E_{\mathrm{r}}+2\right)\right)=0 \quad (\mathrm{IV})$$

Similarly to condition (II), it can be proved that the condition (III) is violated (not shown). Condition (IV) includes a fourth degree polynomial. Importantly, note all its coefficients are the parameters of the recurrent excitatory ($\mathrm{E}\rightarrow\mathrm{E}$) synapses. Here we do not further treat this condition analytically, but instead point to simulations (see Results): For the fixed values that we chose for $\mathrm{E}\rightarrow\mathrm{E}$ STP parameters, i.e. U_{EE}, $\tau_{\mathrm{r_{EE}}}$ and $\tau_{\mathrm{f_{EE}}}$ (see Table 1), depending on the strength of J_{EE} and G_{EE} the quasi E_{r}-nullcline can have either a monotonically decaying or a non-monotonic shape for positive activity rates. For instance, the latter may possess one maximum (i.e. a concave shape; see Results), where this extremum must be an admissible (i.e. real positive) root of condition (IV). We name any of such roots, which are readily numerically computable, a "sign-switching rate", $E_{\mathrm{r}}^{\mathrm{SSR}}$.

Slopes of Frozen STP-RNN's nullclines

The slopes of the E_{r}- and I_{r}-nullclines in a STP-RNN with synaptic efficacies frozen at the FP, i.e. in its corresponding Frozen STP-RNN ($slope_{\mathrm{E}}^{\text{STP-frz}}$ and $slope_{\mathrm{I}}^{\text{STP-frz}}$), can be computed by adapting equation (13). Since in this network the synaptic efficacies are kept fixed at the FP, we set the terms $d\psi_{\mathrm{EE}}\left(E_{\mathrm{r}}\right)/dE_{\mathrm{r}}$, $d\psi_{\mathrm{EI}}\left(I_{\mathrm{r}}\right)/dI_{\mathrm{r}}$, $d\psi_{\mathrm{IE}}\left(E_{\mathrm{r}}\right)/dE_{\mathrm{r}}$, and $d\psi_{\mathrm{II}}\left(I_{\mathrm{r}}\right)/dI_{\mathrm{r}}$ (see equation (13)) all equal to zero. We then have:

$$\begin{aligned} slope_{\mathrm{E}}^{\text{STP-frz}}&=\frac{dI_{\mathrm{r}}}{dE_{\mathrm{r}}}=\frac{G_{\mathrm{E}}J_{\mathrm{EE}}^{\mathrm{FP}}-1}{G_{\mathrm{E}}J_{\mathrm{EI}}^{\mathrm{FP}}} \\ slope_{\mathrm{I}}^{\text{STP-frz}}&=\frac{dI_{\mathrm{r}}}{dE_{\mathrm{r}}}=\frac{G_{\mathrm{I}}J_{\mathrm{IE}}^{\mathrm{FP}}}{1+G_{\mathrm{I}}J_{\mathrm{II}}^{\mathrm{FP}}} \end{aligned} \quad (16)$$

Based on these slopes we can conclude that, for positive activity rates, the I_{r}- nullcline remains always positive in the $E_{\mathrm{r}}\text{-}I_{\mathrm{r}}\text{-plane}$, while the slope of the excitatory one can be either negative or positive, depending on the values of $J_{\mathrm{EE}}^{\mathrm{FP}}$ and G_{EE} (i.e. $\mathrm{E} \rightarrow \mathrm{E}$ synaptic parameters). This indicates that none of the Frozen STP-RNN's nullclines can have a sign-switching rate, since their slopes defined at the FP are activity independent. However, the sign of $slope_{\mathrm{E}}^{\text{STP-frz}}$ implicitly depends on the E-activity rate of the FP at which we freeze the synaptic efficacies. This is because the value of $J_{\mathrm{EE}}^{\mathrm{FP}}$ (and thus the sign of $G_{\mathrm{E}} J_{\mathrm{EE}}^{\mathrm{FP}} - 1$) depends on E_{r} level at the FP (equations (5) and (9)). When considering all potential Frozen STP-RNNs in different FPs in a $E_{\mathrm{r}}\text{-}I_{\mathrm{r}}\text{-plane}$, at least one potential E_{r} level may exist around which the Frozen STP-RNNs have $slope_{\mathrm{E}}^{\text{STP-frz}}$ with different signs. Such a rate or rates can be readily computed as the root or roots of the $G_{\mathrm{E}} J_{\mathrm{EE}}^{\mathrm{FP}} - 1$. We name any of such a root a "pseudo sign-switching rate", $E_{\mathrm{r}}^{\mathrm{pSSR}}$. The existence of pseudo sign-switching rates plays a central role in determining the operating regimes of STP-RNNs; see below.

Characterization of operating regimes

To study the developmental changes in the operating regimes of immature networks we first need to define and classify these operating regimes. One established theoretical definition of operating regimes, proposed for adult RNNs, classifies these regimes as inhibition-stabilized networks (ISNs) vs. Non-ISNs [1,8]. This definition takes all recurrent ($\mathrm{E} \rightarrow \mathrm{E}$ and $\mathrm{I} \rightarrow \mathrm{I}$) and reciprocal feedback ($\mathrm{E} \rightarrow \mathrm{I}$ and $\mathrm{I} \rightarrow \mathrm{E}$) synaptic connections into consideration, consistent with cortical connectivity patterns. The ISN regime (vs. Non-ISN) is thought to provide a general strategy for supporting complex computations while avoiding instabilities [11], and to be the substrate for several cortical phenomena such as the so-called "paradoxical effect" (increasing the excitation to the I-population decreases its activity rate [1,8]) and the balanced amplification mechanism [7]. For instance, [8] reported that an ISN is the only architecture that can explain their intra-cellular recordings in the context of center-surround effects in adult cat V1. Accordingly, we decided to use this well-established classification to study in what regime the postnatal developing networks operate.

For definitions, we rely on previous work [1,8] that used a stability analysis framework of a Static-RNN-type model at the FP. The authors defined three criteria for discriminating between ISNs and non-ISNs at the FP: (**A**) Excitatory instability: For the inhibitory activity rate fixed at the FP, the recurrent excitation is strong enough to render the E-population intrinsically unstable; depending on initial conditions, its activity either dies off or saturates at a high activity rate. (**B**) Excitatory stability: In contrast to (**A**), the E-population is stable *per se*, i.e. even with a feedback inhibition fixed at its level at the FP. (**C**) Overall stability: The dynamic feedback inhibition to the E-population is strong enough to stabilize the whole network activity. A network operating under (**A**) and (**C**) criteria is an ISN, while a network operating under (**B**) and (**C**) criteria is a Non-ISN. This implies that both ISN and Non-ISN regimes are stable, where they differ only in terms of the excitatory stability behavior. These criteria were already derived for RNNs lacking STP effects (i.e. Static-RNN-type models) [1,8]. However, the immature networks considered in this study are STP-RNN-type models. Hence, here our main focus will be to derive the criteria underlying these regimes for STP-RNNs.

Technically, these three stability criteria can be investigated within linear stability theory (similarly to refs [1,8]; see also refs [2,11]), which in general can determine the stability of the FPs. We found in our preliminary analysis that applying this theory to STP-RNN (10D; see equation (4)) leads to rather complicated expressions, which make the derivation of analytical formalism difficult. Therefore, we reasonably simplified these calculations for STP-RNN by exploiting the following facts. First, linear stability theory is applied in vicinity of the FP of interest. Second, at a FP all network variables are at their steady states (with the stability to be determined). Third, the steady states of the STP variables are only functions of activity variables (see equation (5)). Fourth, the time constants of the activity variables (τ_{E} and τ_{I}; usually some milliseconds) are much smaller than those of STD and STF ($\tau_{\mathrm{r}_{\mathrm{ij}}}$ and $\tau_{\mathrm{f}_{\mathrm{ij}}}$; usually several tens to hundreds of milliseconds). Accordingly, to study the stability of the network based on its activity in vicinity of the FP of interest we use a timescales separation technique (see also refs [9,12]): We assume that following a small perturbation used to perturb the network relaxed at the FP of interest, the slower variables (i.e. x_{ij} and u_{ij}) do not change considerably as compared to faster variables (i.e. E_{r} and I_{r}), and thus can be assumed to be fixed at their steady states at the FP (i.e. $x_{\mathrm{ij}}^{\mathrm{FP}}$ and $u_{\mathrm{ij}}^{\mathrm{FP}}$). Effectively, this assumption will turn the network into a STP-RNN with frozen synaptic efficacies at the FP of interest (i.e. a Frozen STP-RNN), as we have already defined in equation (9).

In the following we use this 2D system of equations for deriving the criteria for network regimes and FP-domains (see below). We found that this system, when sub-threshold modes were also considered (see "Sub-threshold networks section" below), provides a reliable approximation to the stability behavior of full system (i.e. 10D; equation (4)) at the FPs, as we checked it by applying the linear stability analysis numerically to the full systems at all FPs of interest (see Results).

To apply linear stability theory, we first linearize the Frozen STP-RNN (equation (9)) around the FP of interest: We take the first-order Taylor expansion of the right-hand side of its ODEs and evaluate the derivatives at the FP. We express the linearized dynamics of the system in matrix form:

$$\tau \frac{d}{dt}\mathbf{A} = -\mathbf{T}^{-1}\left(\mathbf{1}\text{-}\mathbf{J}_{\mathrm{lnr}}^{\text{STP-frz}}\right)\mathbf{A} \quad (17)$$

with $\mathbf{A} = \begin{pmatrix} E_r \\ I_r \end{pmatrix}$, $\mathbf{1} = \begin{pmatrix} 1 & 0 \\ 0 & 1 \end{pmatrix}$, $\mathbf{T} = \begin{pmatrix} 1 & 0 \\ 0 & \alpha \end{pmatrix}$ for some positive constant α, where $\tau_{\mathrm{I}} = \alpha\tau$ and $\tau = \tau_{\mathrm{E}}$, and the linearized (around the FP of interest) connectivity matrix $\mathbf{J}_{\mathrm{lnr}}^{\text{STP-frz}}$, which is obviously equal to $\mathbf{J}^{\text{STP-frz}}$ (Supplementary Table 1). Note that the values of the $\mathbf{J}_{\mathrm{lnr}}^{\text{STP-frz}}$ elements differ from one FP to another as they depend on the activity rates (equation (5)). For the feedback inhibition to the E-population ($\mathrm{I} \rightarrow \mathrm{E}$ connection) fixed at its level at the FP, the upper-right element in these matrix is set to zero. For the sake of clarity, we use the additional subscript "$\backslash\mathrm{EI}$" to discriminate these incomplete connectivity matrix from the original one; i.e. $\mathbf{J}_{\mathrm{lnr}\backslash\mathrm{EI}}^{\text{STP-frz}}$. We further define the corresponding full-coefficient ($\mathbf{M}_{\mathrm{lnr}}^{\text{STP-frz}} = -\mathbf{T}^{-1}\left(\mathbf{1} - \mathbf{J}_{\mathrm{lnr}}^{\text{STP-frz}}\right)$) and incomplete-coefficient ($\mathbf{M}_{\mathrm{lnr}\backslash\mathrm{EI}}^{\text{STP-frz}} = -\mathbf{T}^{-1}\left(\mathbf{1} - \mathbf{J}_{\mathrm{lnr}\backslash\mathrm{EI}}^{\text{STP-frz}}\right)$) matrices, at the FP.

Following linear stability theory, we now study the stability behavior of the network around its FP, based on the eigenvalues of its corresponding Jacobian matrix (here, our coefficient matrices): The system is (asymptotically) stable at the FP if necessarily all the eigenvalues have strictly negative real parts, where the existence of at least one eigenvalue with positive real part implies that the FP is unstable. Accordingly, to satisfy the excitatory instability (criterion **A**, see above) at least one of the two eigenvalues of the network's incomplete-coefficient matrix (i.e. $\mathbf{M}_{\mathrm{lnr\backslash EI}}^{\text{STP-frz}}$), i.e. λ_1 and λ_2, must have a positive real part, see also ref. [8]. In contrast, the excitatory stability (criterion **B**) requires these two eigenvalues to have real parts less than zero. The overall stability (criterion **C**) requires both eigenvalues of the $\mathbf{M}_{\mathrm{lnr}}^{\text{STP-frz}}$ to have real parts less than zero. Using the Routh-Hurwitz criteria we restate this criterion as having both the determinant and the trace operators of the negative of the full-coefficient matrix (i.e. $-\mathbf{M}_{\mathrm{lnr}}^{\text{STP-frz}}$) positive (see also ref. [8]).

For a Frozen STP-RNN at the FP, we have $\lambda_1 = G_{\mathrm{E}} J_{\mathrm{EE}}^{\mathrm{FP}} - 1$ and $\lambda_2 = -\left(1 + G_{\mathrm{I}} J_{\mathrm{II}}^{\mathrm{FP}}\right) / \alpha$. Since λ_2 is always negative, hence $G_{\mathrm{E}} J_{\mathrm{EE}}^{\mathrm{FP}} > 1$ (i.e. $\lambda_1 > 0$) and $G_{\mathrm{E}} J_{\mathrm{EE}}^{\mathrm{FP}} < 1$ (i.e. $\lambda_1 < 0$) fulfill the excitatory instability and excitatory stability criteria, respectively. Considering equation (16), the excitatory instability indicates $slope_{\mathrm{E}}^{\text{STP-frz}} > 0$ and excitatory stability indicates $slope_{\mathrm{E}}^{\text{STP-frz}} < 0$, at the FP. The determinant condition can also be shown to yield $slope_{\mathrm{I}}^{\text{STP-frz}} > slope_{\mathrm{E}}^{\text{STP-frz}}$, at the FP. The trace condition yields $1 + G_{\mathrm{I}} J_{\mathrm{II}}^{\mathrm{FP}} > \alpha\left(G_{\mathrm{E}} J_{\mathrm{EE}}^{\mathrm{FP}} - 1\right)$ at the FP, which holds if α is sufficiently small; for more details see ref. [8].

Similar results have been already obtained by previous studies based on the Static-RNNs (e.g. see equation (3)), which neglected the effect of STP [1,8]. But, for a STP-RNN, the synaptic efficacies of the corresponding Frozen STP-RNNs (equation (9)) can have different values at different FPs (equation (5)). In practice, this implies that, in contrast to a Static-RNN (equation (3)), a STP-RNN has two distinct operational features (see Results): i) its operating regime at a FP is dependent on the activity rate levels at that FP (see previous paragraph), ii) its operating regimes, e.g. the ISN and Non-ISN regimes, may be co-existing rather than being mutually exclusive regimes (e.g., in E_{r}-I_{r}-plane; see "Operational FP-domains" section below); this case can happen in the presence of a pseudo sign-switching rate $E_{\mathrm{r}}^{\mathrm{pSSR}}$ (see the text below equation (16)).

Sub-threshold networks

All analyses above were done for a supra-threshold network, where both E- and I-populations are in supra-threshold mode (see equation (2)). We now generalize these analyses to the sub-threshold modes (see equation (2)): i) sub-threshold network ($h_{\mathrm{E}} \le \theta_{\mathrm{E}}$ and $h_{\mathrm{I}} \le \theta_{\mathrm{I}}$), ii) sub-threshold E-population ($h_{\mathrm{E}} \le \theta_{\mathrm{E}}$ and $h_{\mathrm{I}} > \theta_{\mathrm{I}}$), and iii) sub-threshold I-population ($h_{\mathrm{E}} > \theta_{\mathrm{E}}$ and $h_{\mathrm{I}} \le \theta_{\mathrm{I}}$). The equation (4) for the STP-RNN (similarly, the equation (9) for a Frozen STP-RNN) can be re-written to derive the equations governing the network dynamics in these modes. This can be simply done by setting $G_{\mathrm{E}} = G_{\mathrm{I}} = 0$ for a sub-threshold network, $G_{\mathrm{E}} = 0$ for a sub-threshold E-population mode, and $G_{\mathrm{I}} = 0$ for a sub-threshold I-

population mode. Similarly, the steady state values and the resulting steady state connectivity matrices for these networks can also be obtained from those listed in Supplementary Table 1.

In mode (i), the only FP of the network is the origin (i.e. $E_r = I_r = 0$ Hz) in the E_r-I_r-plane. At this FP, the network is essentially stable. This can be shown by computing the eigenvalues of the corresponding full-coefficient matrix ($\mathbf{M}_{\mathrm{lnr}}^{\text{STP-frz}}$) of this mode, at the FP: $\lambda_1^{(i)} = -1$ and $\lambda_2^{(i)} = -1/\alpha$ which are both always negative; the upper-index indicates the index of the sub-threshold mode. At this FP, the network operates necessarily as a Non-ISN, as there are no feedback connections to the populations (and in particular, there is no $\mathrm{I} \to \mathrm{E}$ connection).

In mode (ii), E_r-nullcline (and quasi E_r-nullcline) lies on $E_r = 0$ Hz line in the E_r-I_r-plane. A FP located on this vertical branch is always stable. This is because, at a FP in this mode we have $\lambda_1^{(ii)} = -1$, and $\lambda_2^{(ii)} = -\left(1 + G_{\mathrm{I}} J_{\mathrm{II}}^{\mathrm{FP}}\right)/\alpha$, which are always negative. Obviously, the corresponding $\mathbf{M}_{\mathrm{lnr\backslash EI}}^{\text{STP-frz}}$ and $\mathbf{M}_{\mathrm{lnr}}^{\text{STP-frz}}$ matrices of this mode (at the FP) are equal, and thus the same eigenvalues as $\lambda_1^{(ii)}$ and $\lambda_2^{(ii)}$ can be obtained based on $\mathbf{M}_{\mathrm{lnr\backslash EI}}^{\text{STP-frz}}$. Since both $\lambda_1^{(ii)}$ and $\lambda_2^{(ii)}$ are negative (criterion **B**), so we can conclude that the Frozen STP-RNN will operate as a Non-ISN at the FP located on the vertical branch at $E_r = 0$ Hz. Moreover, note that in this mode, similarly to the supra-threshold networks, the slope of I_r-nullcline at such a FP is positive. This can be readily shown by adapting our analyses in "Phase plane and nullclines" section to this mode; the $slope_{\mathrm{I}}^{\text{STP-frz}}$ in this mode is equal to that in equation (16).

In mode (iii), I_r-nullcline (and quasi I_r-nullcline) lies on $I_r = 0$ Hz line in the E_r-I_r-plane. A FP located on this horizontal branch can be either stable or unstable. This is because, while at a FP in this mode we have $\lambda_2^{(iii)} = -1/\alpha$, $\lambda_1^{(iii)} = G_{\mathrm{E}} J_{\mathrm{EE}}^{\mathrm{FP}} - 1$. Accordingly, $\lambda_1^{(iii)}$ can be either positive or negative; if negative, the FP is stable, otherwise the FP is unstable. Similarly to mode (ii), the eigenvalues based on the corresponding $\mathbf{M}_{\mathrm{lnr\backslash EI}}^{\text{STP-frz}}$ of this mode (at the FP) are the same as $\lambda_1^{(iii)}$ and $\lambda_2^{(iii)}$. Since $\lambda_2^{(iii)}$ is always negative, therefore depending on the sign of $\lambda_1^{(iii)}$ at the FP located on this horizontal branch, the network can be either unstable (if $\lambda_1^{(iii)} > 0$) or a Non-ISN (if $\lambda_1^{(iii)} < 0$, criterion **B**); note the condition $\lambda_1^{(iii)} > 0$, required for a ISN regime (criterion **A**), violates the overall stability condition of network. Similarly, the E_r-nullcline can have either a negative or positive slope sign at the FP, depending on the sign of $\lambda_1^{(iii)}$; the $slope_{\mathrm{E}}^{\text{STP-frz}}$ is equal to that in equation (16).

Summary of rules for operating regimes

We can now determine two simple rules to distinguish between ISN and Non-ISN regimes in the positive ranges of activity rates in the E_r-I_r-plane. Rule I: For sufficiently small values of α, at the FP, a STP-RNN (equation (4)) operates as an ISN if $slope_{\mathrm{I}}^{\text{STP-frz}} > slope_{\mathrm{E}}^{\text{STP-frz}} > 0$ or as a Non-ISN if $\left(slope_{\mathrm{I}}^{\text{STP-frz}} > 0\right) > \left(slope_{\mathrm{E}}^{\text{STP-frz}} < 0\right)$. Rule II: at the FP, the STP-RNN operating neither as ISN nor Non-ISN is unstable. This is equivalent to saying, at the FP, the STP-RNN has overall *instability* if

$slope_{\mathrm{E}}^{\text{STP-frz}} > slope_{\mathrm{I}}^{\text{STP-frz}} > 0$ or the inhibitory population has a sufficiently slow time constant relative to the excitatory one. Note that when determining these rules we also considered the fact that the I_{r}-nullcline has always positive slope in the E_{r}-I_{r}-plane for positive activity rates (see equation (16)). Besides, we determine the following rules for the FPs locating on the borders of the E_{r}-I_{r}-plane (i.e. at $E_{\mathrm{r}} = 0$ and/or $I_{\mathrm{r}} = 0$ Hz), belonging to the sub-threshold modes (see previous section). Rule III: At the origin ($E_{\mathrm{r}} = I_{\mathrm{r}} = 0$ Hz), the STP-RNN operates as a Non-ISN. Rule IV: At the FP located on the vertical branch of the E_{r}-nullcline (or quasi E_{r}-nullcline) at $E_{\mathrm{r}} = 0$, the STP-RNN operates as a Non-ISN. Rule V: At the FP located on the horizontal branch of the I_{r}-nullcline (or quasi I_{r}-nullcline) at $I_{\mathrm{r}} = 0$ (but $E_{\mathrm{r}} \neq 0$), the STP-RNN is unstable if $slope_{\mathrm{E}}^{\text{STP-frz}} > 0$ or is a Non-ISN if $slope_{\mathrm{E}}^{\text{STP-frz}} < 0$.

Furthermore, one can deduce from Rule II and Rule V that an ISN (but not a Non-ISN) is prone to overall instability, because of the intrinsic instability of its E-population's activity (i.e. $slope_{\mathrm{E}}^{\text{STP-frz}} > 0$ at the FP). Considering this point, together with the expressions we derived above for λ_1 and $\lambda_1^{(\text{iii})}$ (see also equation (5)), we stress the key role of $\mathrm{E} \to \mathrm{E}$ synapses in governing the overall instability, where weak $\mathrm{E} \to \mathrm{E}$ synapses can effectively diminish or remove instability. The second source of overall instability effectively depends on the ratio of the synaptic time constants τ_{E} and τ_{I} (i.e. α; see the trace condition $1 + G_{\mathrm{I}} J_{\mathrm{II}}^{\text{FP}} > \alpha \left(G_{\mathrm{E}} J_{\mathrm{EE}}^{\text{FP}} - 1 \right)$ below equation (17)), and can just accompany the potential instability effect caused by $\mathrm{E} \to \mathrm{E}$ synapses. This means that the removal of $\mathrm{E} \to \mathrm{E}$ synapses can effectively eliminate these both sources of overall instability. In addition, note that this conclusion, as well as the five rules (Rule I to Rule V), are based on the assumption that the GABAergic transmission acts on the whole as inhibitory at the network level (i.e. when J_{EI} and J_{II} are positive). Otherwise, at least, $\mathrm{I} \to \mathrm{I}$ synapses will also accompany to the second source of instability (i.e. through the trace condition), where its corresponding contribution will be effectively regulated by α.

Parameterization of postnatal developing networks

In this work, we aimed at studying the developmental changes in immature network dynamics *in vivo*, during the first postnatal month. During this period, these networks undergo dramatic refinements in their biophysical characteristics, including the intrinsic neuronal properties (e.g. membrane resistances), STP properties (e.g. release probabilities), and the effect of GABAergic synaptic transmission on immature neurons [13,14]. The sparsification phenomenon also occurs in this period: the network's spontaneous activity transition from highly synchronized (cluster activity) to more sparse firing patterns [15,16]. Overall, as an example of sensory cortices undergoing such developmental changes in both biophysical characteristics and network dynamics, we consider the visual cortex for which the sparsification is mainly set in around eye-opening [16,17]. Accordingly, to cover the range of postnatal days (P) towards the complete process of sparsification (starting from birth) we study the dynamics of a developing RNN model at four stages of visual cortex which we modeled: P3 (period of physiological blindness), P9 (a few days before eye-opening), P14 (the day after eye-opening) and P20 (a few days after eye-opening); see also refs [16,17].

Since STP mechanisms undergo substantial developmental changes and significantly affect (mainly via strong STD) the dynamics of immature networks [14,18], we consider the developing RNN model as a STP-RNN. For this network we make two simplifications. First, consistent with experimental reports [19] (see also refs [6,20]), we assume changes in synaptic projections are independent of the target population type, where the projections of the same type (GABAergic or glutamatergic) have the same (immature) STP properties, separately. Hence, we use the same values for STP parameters of $\mathrm{E} \to \mathrm{E}$ and $\mathrm{E} \to \mathrm{I}$ connections (U_{E}, $\tau_{\mathrm{r}_{\mathrm{E}}}$, $\tau_{\mathrm{f}_{\mathrm{E}}}$) and of $\mathrm{I} \to \mathrm{I}$ and $\mathrm{I} \to \mathrm{E}$ connections (U_{I}, $\tau_{\mathrm{r}_{\mathrm{I}}}$, $\tau_{\mathrm{f}_{\mathrm{I}}}$). We also assume both glutamatergic connections ($\mathrm{E} \to \mathrm{E}$ and $\mathrm{E} \to \mathrm{I}$) and both GABAergic connections ($\mathrm{I} \to \mathrm{I}$ and $\mathrm{I} \to \mathrm{E}$) have the same absolute synaptic efficacies (J_{E} and J_{I}), separately. This follows as the target-specific synaptic patterns might not, or at least poorly, formed in immature networks. By using these simplifications we aimed at providing a clearer representation of our main findings about the prominent phenomena occurring between P3 to P20 (see Results). However, we stress that: i) for the mathematical framework of our analyses (see above), we made no assumption about the parameter values and thus our analytical (algebraic) results were obtained for a general case, ii) in our preliminary results, we observed that our main findings (see Results) remained mainly intact even without these assumptions. Nonetheless, note that further electrophysiological experiments are required to be done for this postnatal period, in order to provide more detailed distinction and characterization of, e.g. the STP properties and synaptic patterns between different neuron types.

To incorporate the postnatal developmental changes in the parameter values of a STP-RNN we re-parameterized it for each of P3, P10, P14 and P20 stages, separately (see Table 1); based on the currently available experimental findings (mostly from visual cortex). To do this, we made the following assumptions for a network growing during P3 to P20 (see Table 1):

1) The (synaptic) time constants of E- and I-populations (τ_{E} and τ_{I}) decrease profoundly during this period. This is effectively due to the substantial developmental decline in membrane resistance of neurons [14,15]. Recall τ_{E} and τ_{I} are typically some approximations of the decay time constants of glutamatergic and GABAergic postsynaptic responses [4,8].

2) Both receptor-mediated glutamatergic and GABAergic synaptic projections are strongly depressing at an early postnatal age (P3), and become less depressing towards P20. This is based on findings about strong developmental increments in the paired pulse ratios of postsynaptic responses, and the developmental changes in the organization and mechanisms governing the vesicle pools machinery [14,18]. Accordingly, we translated these findings into the reported reductions of release probabilities (U_{E} and U_{I}) and time constants of synaptic depression ($\tau_{\mathrm{r}_{\mathrm{E}}}$ and $\tau_{\mathrm{r}_{\mathrm{I}}}$) and facilitation ($\tau_{\mathrm{f}_{\mathrm{E}}}$ and $\tau_{\mathrm{f}_{\mathrm{I}}}$) mechanisms.

3) GABAergic synapses are on the whole less depressing than glutamatergic ones and this difference increases from P3 to P20 (especially after eye-opening) due to the maturation of the STP mechanisms; e.g. see experimental reports in refs [21,22]. This difference can be seen in the relatively higher values of $\tau_{\mathrm{r}_{\mathrm{E}}}$ and U_{E} as compared to $\tau_{\mathrm{r}_{\mathrm{I}}}$ and U_{I} in Table 1.

4) The thresholds of E- and I-populations activity (θ_{E} and θ_{I}) increase from P3 to P20. In our model these parameters depend effectively on i) background inputs (e.g. driven by neuropil activity [2,4]) and ii) membrane resistance. During P3 to P20, the background activity increases, especially after the onset of sensory transduction [15-17]; e.g. see the developmental increase in the frequency of spontaneous Ca^{2+} waves in refs. [15,16]. In contrast, the membrane resistance profoundly declines during this period [14,15], which, according to Ohm's law, implies that the depolarizing membrane current required for triggering an AP is increased during development [23]. We assume that the overall effect of these developmental changes leads to the experimentally observed increase in the corresponding rheobase currents during this period [14]. Intuitively, this means that the neurons become harder to fire. In terms of the populations activity, this indicates a developmental increase in θ_{E} and θ_{I}. In brief, when considering a single stage, the increase in the background input to a population can be modeled by decreasing its threshold (θ_{E} or θ_{I}), while the decline in membrane resistance can be modeled by increasing that threshold. Moreover, note that we assumed θ_{I} is in general to some extent larger than θ_{E} [2].

5) The effective absolute synaptic efficacy of average excitatory projections (i.e. $G_{\mathrm{E}}J_{\mathrm{E}}$; for simplicity we set $G_{\mathrm{E}} = 1$) increases prior to eye-opening and then starts to decay toward P20. This is mainly based on the findings about the developmental increase (before eye-opening) followed, after eye-opening, by a decrease in the amplitude of the mean unitary (maximal) glutamatergic postsynaptic responses [14,18]. Moreover, at early postnatal days (before eye-opening) these synapses are overexpressed, and there is a high density of glutamatergic receptors [13,24]. The un-silencing of these synapses, and in particular the start of functioning of AMPA receptors at the end of first postnatal week [13,24], may determine the early increase in these responses; see also ref. [25]. However, the further maturation of neuronal mechanisms and organizations after eye-opening tends to reduce the excitatory factors, e.g. due to a downregulation in the number of functional contacts per excitatory synaptic connection [18]; see also ref. [25].

6) The effective absolute synaptic efficacy of average inhibitory projections (i.e. $G_{\mathrm{I}}J_{\mathrm{I}}$; for simplicity we set $G_{\mathrm{I}} = 1$) increases from P3 to P20. In particular, we assigned a very small value to this parameter at P3 (rendering the inhibitory processing remarkably weak), a relatively much higher value at P10, and with more smooth increments toward P20. These changes should underlie the following experimental findings about [13,24,25]: i) the relative paucity of inhibitory synapses in immature networks, ii) the lack of postsynaptic $GABA_B$ receptor-mediated responses until the end of first postnatal week, iii) the shift in activation effect of $GABA_A$ receptors on membrane potential of neonatal neurons, namely from partially depolarizing (during first postnatal week) to its classical hyperpolarizing effect, and iv) strengthening of the inhibitory effect during the developing period. Regardless of iii), we still consider the overall GABAergic transmission to be inhibitory at the *network level*, even during first postnatal week; consistent with recent *in vivo* experiments [26,27]. However, for completeness, we also separately consider the case that GABAergic transmission is on the whole excitatory at the *network level* (see Supplementary Fig. 3).

Additional parameters and definitions

In the following, we define a number of numerical parameters, and describe some specific properties, which are relevant for the obtained results.

Operational FP-domains

For a STP-RNN, we partition the E_{r}-I_{r}-plane into different domains of FPs (FP-domains), where each FP-domain belongs to a different operating regime (see Results). For instance, the FP-domain of an ISN contains the steady states (i.e. FPs) at which the network could operate as an ISN. Similarly, we can define the FP-domain of Non-ISN and unstable regimes. To plot these domains (see Results) we utilized our analytically driven (algebraic) expressions for each regime (e.g. see the "Characterization of operating regimes" section).

Moreover, we also numerically compute the two-dimensional integration over the area of each FP-domain (AOD; area of domain), by using a sparse grid rule (i.e. the fully symmetric integration rule) as implemented in Mathematica (version 9). Note that for all FP-domains, the integration limits do not exceed the minimum (here, always the origin) and maximum E- and I-activity rates ($[E_{\mathrm{r}}^{\max}, I_{\mathrm{r}}^{\max}]$), which are determined by the desired phase plane (see Results). In addition to the area of ISN (AOD_{ISN}), and Non-ISN ($AOD_{\mathrm{Non\text{-}ISN}}$) FP-domains, we also compute $AOD_{\mathrm{ISN/Unstable}} = AOD_{\mathrm{ISN}} / AOD_{\mathrm{Unstable}}$; clearly, this ratio measures the AOD_{ISN} vs. AOD_{Unstable}, and vice versa. For instance, an increase in $AOD_{\mathrm{ISN/Unstable}}$ indicates that within the E-activity ranges where both ISN and unstable regimes co-exist (although at different I-activity ranges; see Results), the unstable FP-domain has been replaced by ISN FP-domain in the E_{r}-I_{r}-plane. In other words, the increase in $AOD_{\mathrm{ISN/Unstable}}$ shows an effective increase in ISN FP-domain in parallel to an effective decrease in unstable FP-domain, and vice versa.

Mono-stable and bi-stable trajectories

Any particular solution of a dynamical system (as a function of time) sketched in the phase plane is called the trajectory of that solution; or simply the trajectory. By inspecting the trajectory one can see whether the underlying solution converges to a FP as time increases. Here, we briefly describe two types of the trajectories, i.e. mono-stable and bi-stable, which we use later when explaining the emergence of cluster activity (see Results). A mono-stable trajectory appears in a mono-stable system, where the trajectory is initiated and terminated at the only stable FP of the system. In contrast, a bi-stable trajectory appears in bi-stable systems, where the trajectory connects the two stable FPs of the system. For a description of these trajectories see, for example, ref. [28].

Population spike

A population spike (PS) reflects the near-synchronous activation of many neurons [12,29]. During a PS in excitatory (PS_E) or inhibitory (PS_I) populations, the population activity diverges, makes an overshoot, and then returns to an asynchronous activity level. A PS with larger amplitude indicates that more neurons participated in the underlying synchronous event [12,29]. For a recurrent network of E- and I-populations (RNN), this event underlies cluster activity (PS_{net}) which usually involves both populations (i.e. it is a combination of PS_E and PS_I).

The cluster activity size is defined as the number of E- and I-neurons which become active during the cluster. We can qualitatively estimate this quantity from the network sum activity ($A_{\rm sum} = E_{\rm r} + I_{\rm r}$). This follows as in our Wilson-Cowan-type model $E_{\rm r}$ and $I_{\rm r}$ denote the average activity (in hertz) of homogenous E- and I-populations at any given time, which are equivalent to their instantaneous population firing rates (i.e. the number of active neurons in a certain short time interval, in the corresponding population); see refs [2,12]. We define the cluster activity size as $PS_{\rm net}^{\rm amp} = \omega \times \left(A_{\rm sum}^{\rm amp} - A_{\rm sum}^{0} \right)$, where $A_{\rm sum}^{\rm amp}$ and $A_{\rm sum}^{0}$ are the maximal activity during the cluster activity and the preceding activity, respectively, and ω is a scaling factor (in units of [Hz^{-1}]) to convert the activity rate to an approximate number of activated neurons during cluster activity. The veridical value of ω depends on the number of network neurons, which is not defined explicitly for a mean-field model. For simplicity, we set $\omega = 1$ for all stages throughout the paper, so we can compare different cluster activity sizes qualitatively: a larger value of $PS_{\rm net}^{\rm amp}$ (a dimensionless parameter) implies that more E- and I-neurons participated in the underlying cluster activity [12,29].

Similarly, the size of PS_E or PS_I can be defined ($PS_{\rm E}^{\rm amp}$ and $PS_{\rm I}^{\rm amp}$). Note that the cortical neural networks are typically considered to be comprised of 20% GABAergic and 80% glutamatergic neurons. Accordingly, for example, a $PS_{\rm I}^{\rm amp} > PS_{\rm E}^{\rm amp}$ can imply that a higher fraction of GABAergic neurons (out of 20%) than the glutamatergic neurons (out of 80%) were active during a cluster activity.

Moreover, we measure the duration of a cluster activity as its termination time minus its onset time. Here, for each cluster activity we estimate the onset time as the arrival time of perturbation (see Results), and the termination as the time that $A_{\rm sum}$ falls below $A_{\rm sum}^{\rm FP} + 1$ Hz; where, $A_{\rm sum}^{\rm FP}$ is the sum activity at the FP achieved after the perturbation.

Simulations

All results described in this study have been implemented as Matlab (MathWorks) and Mathematica codes. The integration time-step size for simulations is set to 0.001 s.

Supplementary Figure Legends

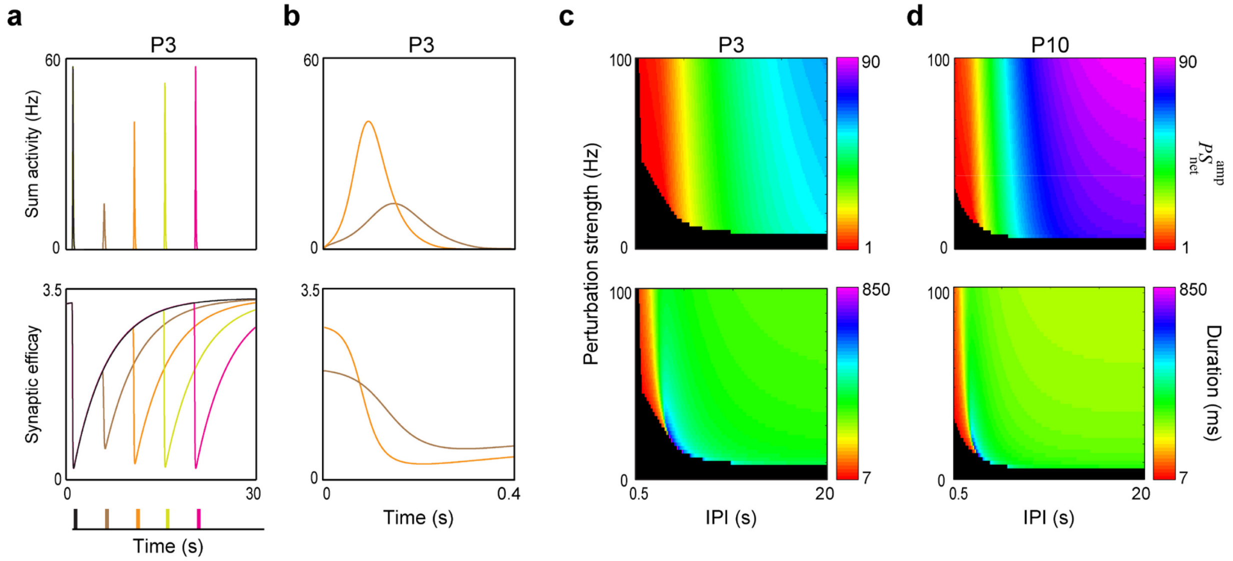

Supplementary Figure 1

The variability in spatiotemporal characteristics of postnatal spontaneous cluster activities. (**a**) The effect of synaptic recovery from a depressing state (lower panel) on size of cluster activities (upper panel) at P3. Following the first cluster activity (black line) elicited by a perturbation input applied to the E-population, a new single perturbation with the same strength (30 Hz) is applied and triggers another cluster activity. The second perturbation times are 5 (brown), 10 (orange), 15 (grey) and 20 s (purple). The plot shows, using four different inter-perturbation intervals (IPI), the sum activity $A_{sum} = E_r + I_r$ (upper panel) and the synaptic efficacy of recurrent excitatory connection $J_E x_E u_E$ (lower panel). (**b**) Zoom-in of the second and third cluster activities in (a), around the time of their perturbation. The plot shows the same effect as (a), but for the duration of cluster activities (upper panel). (**c**) The color-coded matrices show the interaction of perturbation strength and IPIs on size (upper panel) and duration (lower panel) of the cluster activity following the second perturbation, at P3. (**d**) Same as (c), but for P10. For spontaneous activity, the variability in the perturbation strengths and IPIs may reflect the stochasticity in the amplitude and timing of inputs to the network, driven by e.g. spontaneous retinal waves or thalamus [16,26]. It can be seen that cluster activity (upper panel) emerges only when the perturbation strength exceeds some thresholds (black regions, corresponding to the sum activities with maximum size less than 1). The fixed level of the black region at sufficiently long IPIs relates to the amplification-threshold at $I_r = 0$ Hz, in Fig. 3b. These findings are consistent with the experimentally reported all-or-none characteristic of postnatal cluster activities [17,30]. The networks at P3 and P10 were parameterized according to Table 1. The P14 and P20 stages were not shown due to their potential need, after a first perturbation, to receive a large enough inhibitory input to push them from the attractor (Fig. 3c) to the rest state first. However, at these stages, we expect a weaker dependency of cluster activity characteristics on IPIs, as the excitatory synapses recover faster from depression (Table 1).

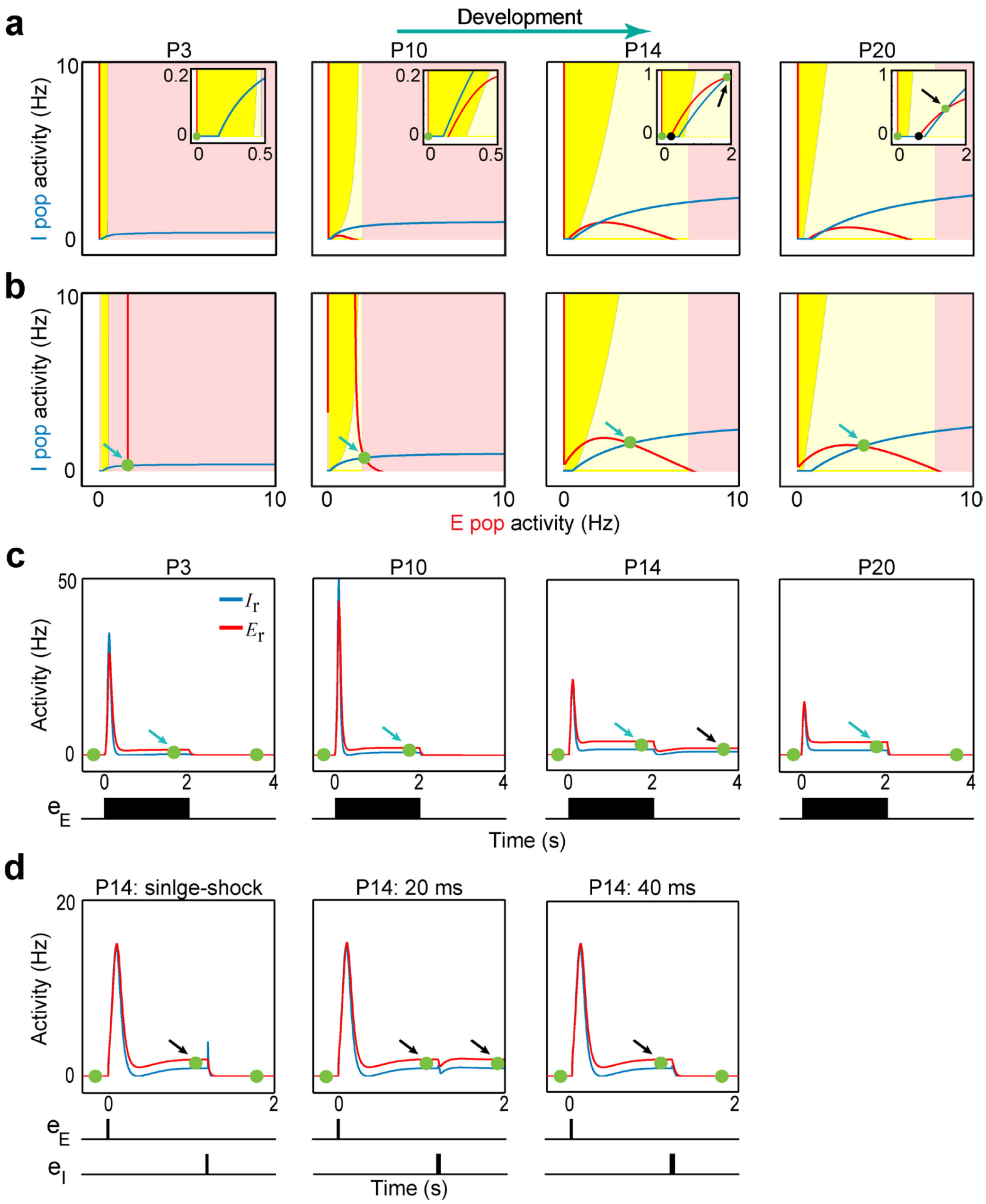

Supplementary Figure 2

Activity properties of developing networks in response to long-lasting inputs. (**a**) The same plots as in Fig 3a; the quasi E_r- and I_r- nullclines were plotted in the absence of external input. (**b**) The emergence of stimulus-evoked attractors in developing networks during development. The same format is used as in (a), but overlaid by the FPs. The same networks as in (a), i.e. when relaxed at their rest states, were stimulated by a constant input ($e_E = 1.3$ Hz) applied to the E-population. The input shifted the quasi E_r- nullcline and a stimulus-evoked attractor (the green dot, pointed to by green arrow) emerged in the Non-ISN (P3 and P10; pink region) and later in the ISN (P14 and P20; light-yellow region) FP-domain. Other combinations of long-lasting inputs to E- and I-populations could be used to set the network's

activity state at other potential, both Non-ISN or ISN stimulus-evoked attractors (not shown). (**c**) Transient feature of stimulus-evoked attractors. The temporal network activities towards the stimulus-evoked attractors in (b) are shown. The new attractor (pointed to by green arrow) is evoked during the input (black bar below the plots). It vanishes after input removal and the network returns to its spontaneous, stable steady state (green dots in (a)). The 2-second input ($e_{\mathrm{E}} = 1.3$ Hz) used here can be due to external stimulation or spontaneous Ca^{2+} waves from the surrounding neuropil of the network [15,16]. (**d**) Enhanced contribution of inhibitory transmission to network activity after eye-opening. For a P14 network, the effect of inhibitory input to the I-population (at $t = 1.2$ s) on the network relaxed at its spontaneous attractor was evaluated. Left panel: an impulse perturbation (single-shock) $e_{\mathrm{I}} = 30$ Hz, Middle panel: $e_{\mathrm{I}} = 1$ Hz for 20 ms, Right panel: $e_{\mathrm{I}} = 1$ Hz for 40 ms. $e_{\mathrm{E}}^{\mathrm{per}} = e_{\mathrm{E}}(t = 0) = 30$ Hz. As observed experimentally for more mature network [31], a strong single-shock (left panel) or a relatively weak but long duration inhibitory input (right panel) were able to terminate the persistent activity and move the network back to its rest state.

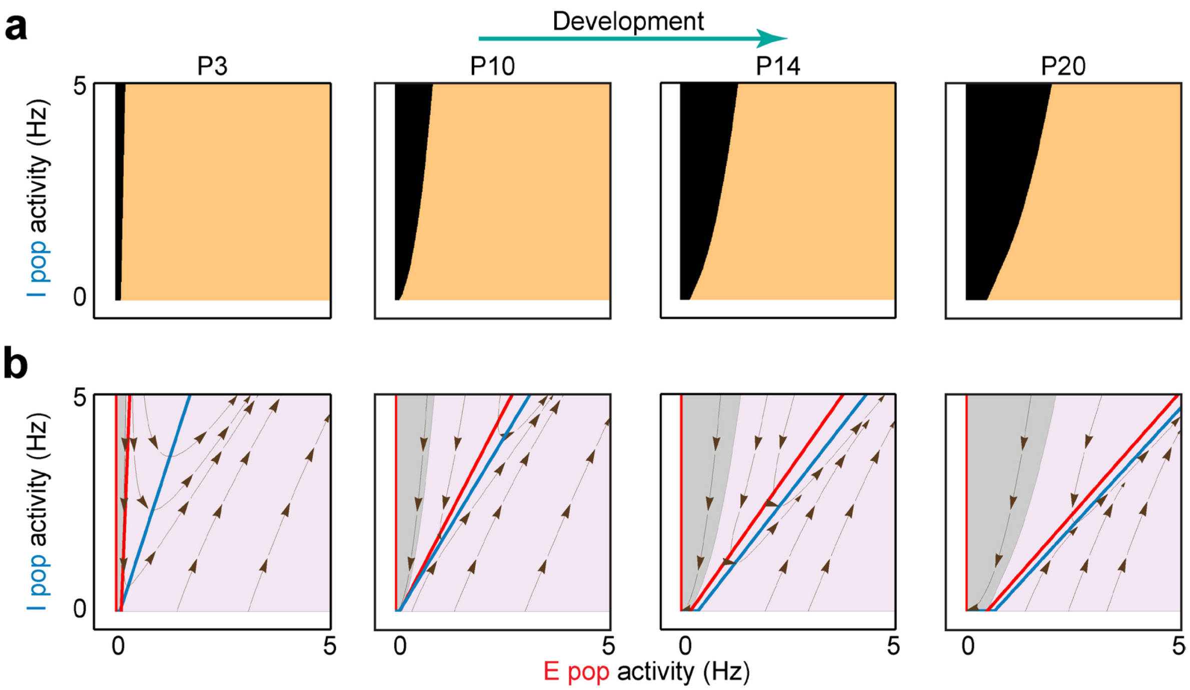

Supplementary Figure 3

Postnatal changes in the amplification-threshold of developing networks. The same format is used as in Figs. 2c and 2d. (**a**) Developmental extension of the non-amplification domain (black region), for the developing STP-RNN. (**b**) Developmental extension of the attraction domain of the rest state (grey region) in the STP-RNNs with synaptic efficacies frozen (Frozen STP-RNN) at the rest state (at $t = -150$ ms, as in Fig. 3b). Note that at each developmental stage, for a network relaxed at the rest state, the non-amplification domain in the STP-RNN shown in (a) and the attraction domain in its corresponding Frozen STP-RNN shown in (b) are very similar in shape.

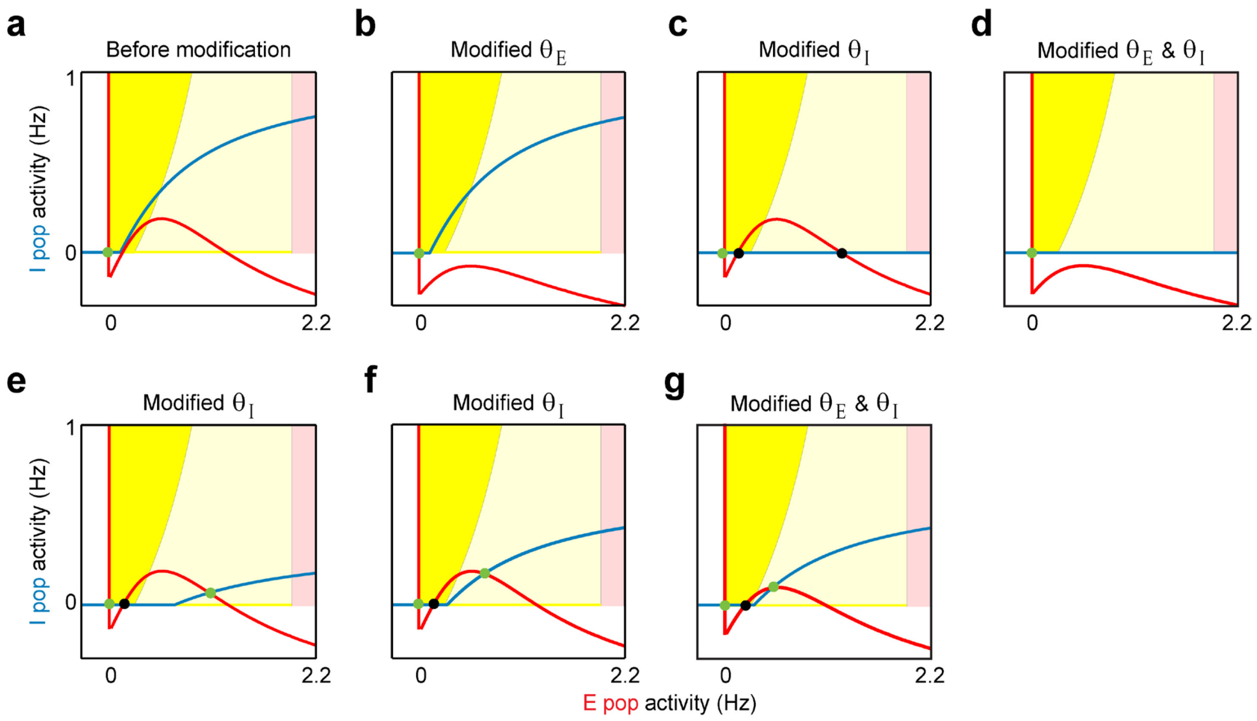

Supplementary Figure 4

Contribution of population activity threshold to the emergence of spontaneous attractors. The same format is used as in Fig. 1b. (**a**) The E_r-I_r-plane of the STP-RNN at P10 based on the parameter values in Table 1, i.e. before the following modification. (**b**) Same as (a) but after substituting the activity threshold of excitatory population θ_E by its value at P20 (1 Hz, Table 1). (**c**) Same as (a) but after substituting the activity threshold of inhibitory population θ_I by its value at P20 (2 Hz, Table 1). (**d**) Same as (a) but after substituting both θ_E and θ_I by their values at P20 (Table 1). (**e**) Same as (a) but after changing θ_I to 1.5 Hz. (**f**) Same as (a) but after changing θ_I to 1 Hz. (**g**) Same as (a) but after changing θ_E and θ_I to 0.6 and 1 Hz. In the model, the values of these parameters are determined by the combined effect of background activity and membrane resistance (see Supplementary Methods). The plots show that these modifications of θ_E and θ_I (increasing relative to their P10 values) result in an overall downward shift in both quasi E- (see (b), (d), and (g)) and I_r- nullclines (see (c)-(g)), relative to (a). Note the emergence of non-origin stable (green dots) and unstable (black dots) FPs due to the modifications of θ_E and θ_I. In sum, the plots show that changes in θ_E and θ_I can contribute to the emergence of spontaneous attractors in developing networks. The negative activity ranges have no physical meaning and are displayed only for better representing the change in quasi nullclines behavior after modification.

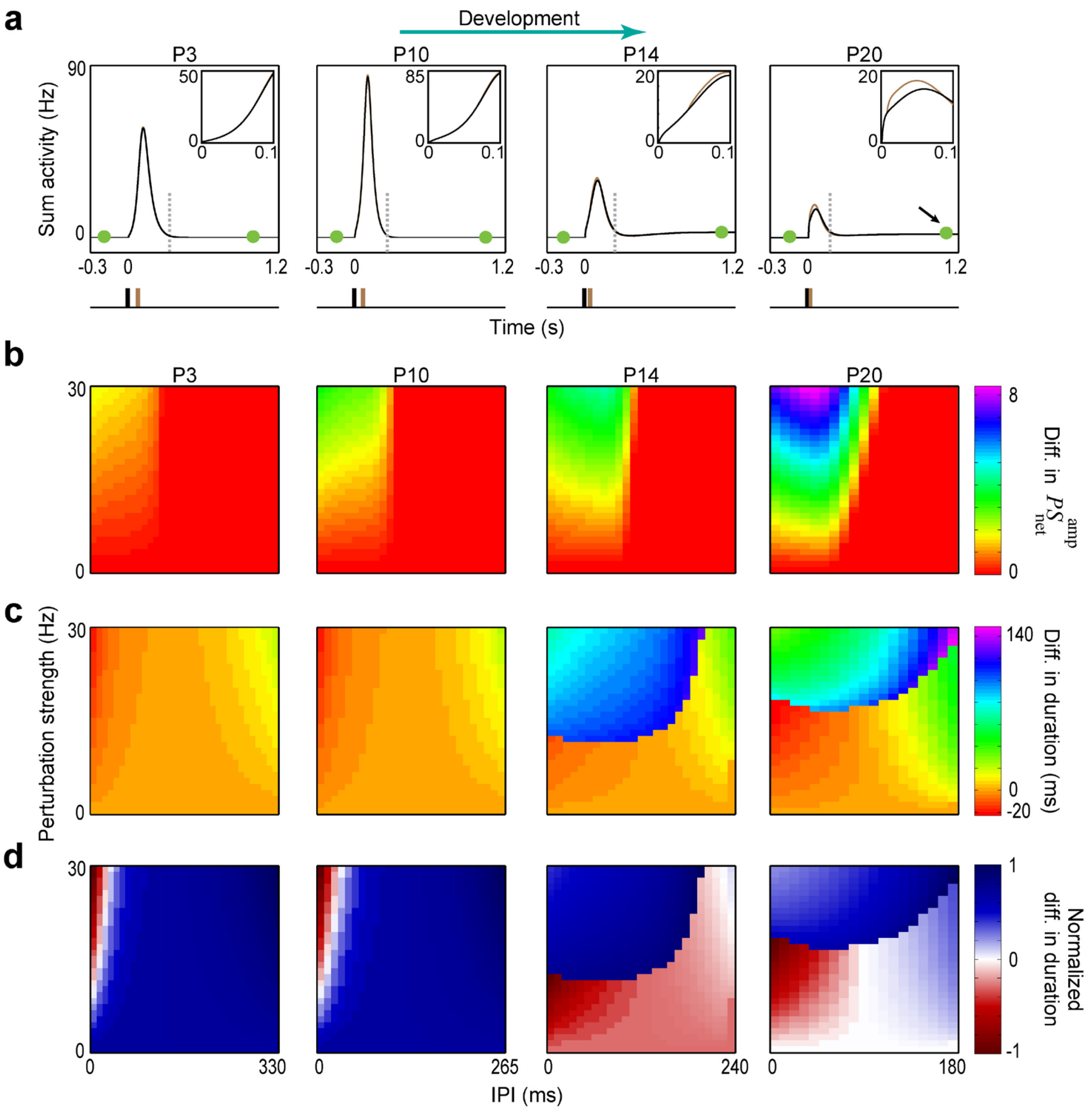

Supplementary Figure 5

Postnatal changes in robustness of cluster activities against interfering inputs. (**a**) The effect of perturbing a cluster activity on its dynamics, during development. Following the first cluster activity (black line) elicited by a perturbation input applied to the E-population at the rest state, a new single perturbation (brown line) is applied when the first cluster activity reaches its half-maximum size. Each plot shows the sum activity $A_{sum} = E_r + I_r$ for the absence (black line) and presence of the second perturbation (brown line). (Insets) Zoom-in of the sum activities at onset of first perturbation. Note that due to the small effect of the perturbation on cluster activity, especially at P3 and P10, the sum activity after second perturbation (brown line) can be barely seen. The perturbation strengths are 30 Hz (black) and 10 Hz (brown). The green dots show the stable FPs in Fig. 3a. (**b**) First row: The effect of different inter-perturbation intervals (IPI) and strength of perturbations, occurring during a cluster activity, on cluster activity size. The colors encode the difference between cluster activity size in the absence and

presence of second perturbation (*presence* minus *absence*). (**c**) Same as (b), but for cluster activity duration. (**d**) Same as (c), but after normalization; the negative and positive differences shown in (c) were normalized to the absolute minimum and maximum differences in cluster activity durations, respectively. Negative and positive values in (c) and (d) indicate the shortening and prolongation of cluster activity duration due to the second perturbation, respectively. In sum, these results show that, as compared to the stage after eye-opening (P14 and P20), the mono-stable cluster activities prior to eye-opening (P3 and P10) are in general more robust against the interfering perturbations in terms of both cluster activity size (see (b)) and duration (see (c) and (d)). Conversely, after eye-opening the networks show more flexible behavior in response to interfering inputs; compare the differences in cluster activity sizes before and after eye-opening in (b). IPIs are limited to the onset (i.e. 0) and duration of cluster activities, in the absence of second perturbation. All four networks were parameterized according to Table 1.

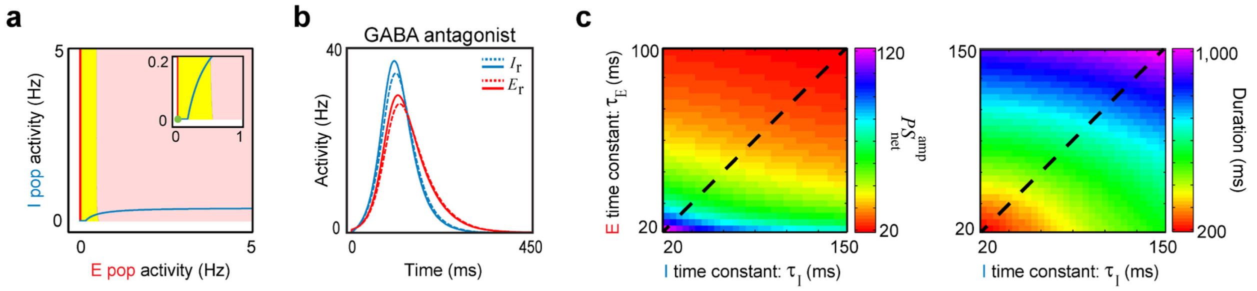

Supplementary Figure 6

Spontaneous stationary and cluster activity properties of a STP-RNN where GABAergic connections at P3 were simulated to be excitatory. (**a**) The same format is used as in Fig. 1b. (**b**) The same format is used as in Fig. 4b. Cluster activities before (solid lines) and after (dashed lines) blocking the GABAergic receptors. This blockage led to a decrease in cluster activity size and prolonged cluster activity duration; $PS_{\text{net}}^{\text{amp}}$: from 67 to 62, Duration: from 318 to 323 ms. (**c**) Effect of different E and I synaptic time constants τ_{E} and τ_{I} on the cluster activity size (first panel) and duration (second panel). The matrix elements below the dashed black line belong to $\alpha > 1$, where $\alpha = \tau_{\text{I}} / \tau_{\text{E}}$. It can be seen that in general parameters τ_{E} and τ_{I} effectively determine the duration of cluster activity, and there is an inverted relationship between their effect on cluster activity size and duration. Simulations are performed for $J_{\text{I}} = -0.1$. The values of all other parameters were set to the same as at the P3 stage in Table 1.

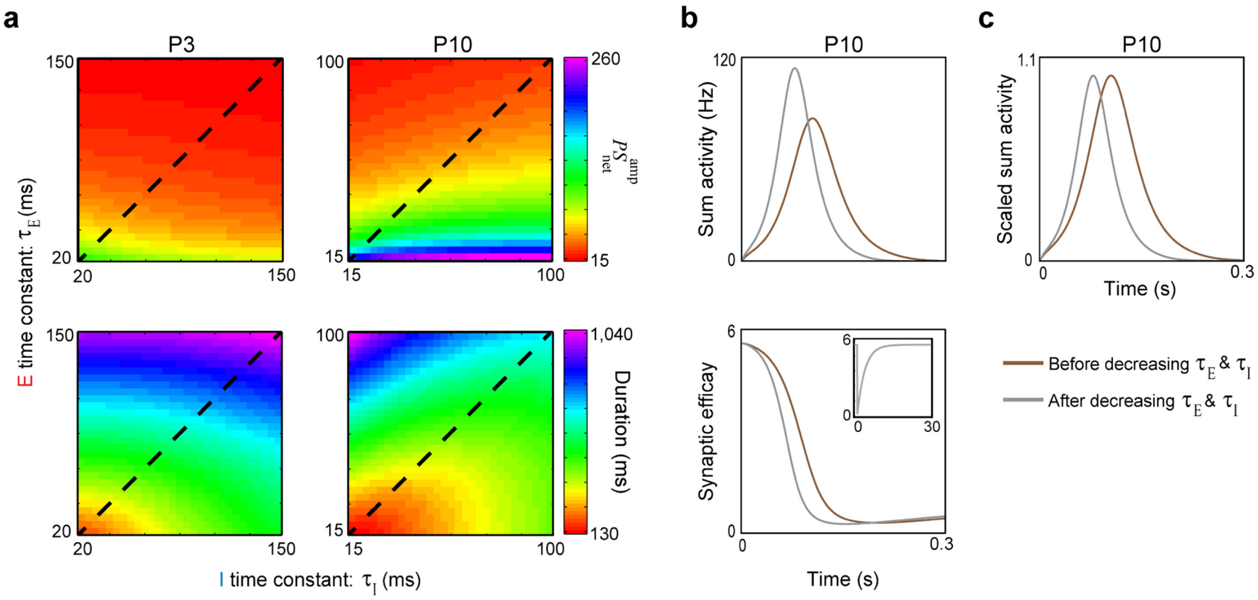

Supplementary Figure 7

Effect of synaptic time constants on postnatal cluster activities before eye-opening. (**a**) The effect of different E and I synaptic time constants τ_E and τ_I on the size (first row) and duration (second row) of cluster activity, at P3 and P10. Note the change in the effect of larger $\alpha > 1$ (the elements below the dashed black line; $\alpha = \tau_I / \tau_E$) and, especially, larger τ_E values on cluster activity size and duration, between these two stages. Overall, it can be seen that parameters τ_E and τ_I have opposing effects on cluster activity size and duration. (**b**) The effect of a 25% decrease in both τ_E and τ_I on cluster activity characteristics at P10. Upper panel: the sum activity ($A_{sum} = E_r + I_r$), Lower panel: synaptic efficacy of recurrent excitatory connection ($J_E\, x_E\, u_E$), Grey lines: after the decrease of τ_E and τ_I, Brown lines: before the decrease of τ_E and τ_I. It can be seen that this modification led to an increase of cluster activity size and a decrease of duration (change from brown to grey lines). (Inset) Zoom-out of the synaptic efficacies. (**c**) The computed sum activities in (b) were scaled to both have a maximum value of 1. Regardless of the size of these cluster activities, the decrease in τ_E and τ_I effectively shrunk the cluster activity duration (grey line). In sum, these results indicate that the values of these parameters (as well as α, see (a)) play an important role in determining the temporal characteristics of cluster activity. The networks at P3 and P10 (before the modification in (b)) were parameterized according to Table 1.

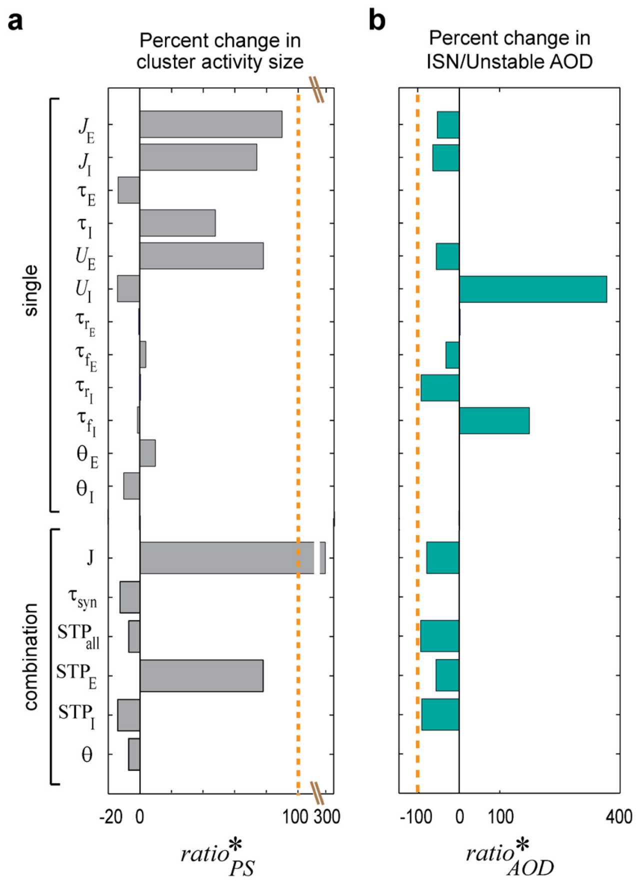

Supplementary Figure 8

Extended analysis for the impact of specific maturational processes on the sparsification process. Similar assessment was done as in Fig. 5, but here we instead, virtually, de-mature the parameter(s) of interest at P20, when transitioning from P10 to P20. To do this we substituted the single or combination of parameters at P20 (Table 1) with their values at P10 (Table 1), and then computed the $ratio^*_\gamma = 100 \times \varpi(\gamma^{res}_{P20} - \gamma_{P20})/(\gamma_{P10} - \gamma_{P20})$, where γ^{res}_{P20} is the value of γ measured after the modification. (**a**) $\gamma = PS^{amp}_{net}$ with $\varpi = +1$. The plotted values of $ratio^*_{PS}$ measure the modification-induced changes in PS^{amp}_{net} relative to the increase when transitioning from P20 to P10. The dashed orange line at $ratio^*_{PS} = +100\%$ indicates the normal amount of increase in PS^{amp}_{net}, as expected when transitioning from P20 to P10. (**b**) $\gamma = AOD_{ISN/Unstable}$ with $\varpi = -1$. The plotted values of $ratio^*_{AOD}$ measure the modification-induced change in $AOD_{ISN/Unstable}$ relative to the decrease when transitioning from P20 to P10. The dashed orange line at $ratio^*_{AOD} = -100\%$ indicates the normal amount of decrease in $AOD_{ISN/Unstable}$, as expected when transitioning from P20 to P10. For computing $AOD_{ISN/Unstable}$, we considered the E_r-I_r-plane plots with $[E^{max}_r, I^{max}_r]$=[10,10] Hz. Parameters combinations are $J = \{J_E, J_I\}$, $\tau_{syn} = \{\tau_E, \tau_I\}$, $STP_{all} = \{STP_E, STP_I\}$, $STP_E = \{U_E, \tau_{r_E}, \tau_{f_E}\}$, $STP_I = \{U_I, \tau_{r_I}, \tau_{f_I}\}$, $\theta = \{\theta_E, \theta_I\}$.

Supplementary Tables

Supplementary Table 1. Stationary components of the network models.

Network type	Steady state value	Steady state connectivity matrix
**Static-RNN**	$E_r = G_E\left(J_{EE}E_r - J_{EI}I_r - \theta_E + e_E\right)$<br>$I_r = G_I\left(J_{IE}E_r - J_{II}I_r - \theta_I + e_I\right)$	$\mathbf{J}^{STC} = \begin{pmatrix} G_E J_{EE} & -G_E J_{EI} \\ G_I J_{IE} & -G_I J_{II} \end{pmatrix}$
**Static-RNN (Frozen STP-RNN)**	$E_r = G_E\left(J_{EE}^{FP}E_r - J_{EI}^{FP}I_r - \theta_E + e_E\right)$<br>$I_r = G_I\left(J_{IE}^{FP}E_r - J_{II}^{FP}I_r - \theta_I + e_I\right)$	$\mathbf{J}^{STP\text{-}frz} = \begin{pmatrix} G_E J_{EE}^{FP} & -G_E J_{EI}^{FP} \\ G_I J_{IE}^{FP} & -G_I J_{II}^{FP} \end{pmatrix}$
**STP-RNN**	$E_r = G_E\left(J_{EE}\,u_{EE}\,x_{EE}\,E_r - J_{EI}\,u_{EI}\,x_{EI}\,I_r - \theta_E + e_E\right)$<br>$I_r = G_I\left(J_{IE}\,u_{IE}\,x_{IE}\,E_r - J_{II}\,u_{II}\,x_{II}\,I_r - \theta_I + e_I\right)$<br>$u_{ij} = \frac{U_{ij}\left(A_j\tau_{f_{ij}} + 1\right)}{A_j U_{ij}\tau_{f_{ij}} + 1}$<br>$x_{ij} = \frac{1}{A_j u_{ij}\tau_{r_{ij}} + 1}$	$\mathbf{J}_{ss}^{STP} = \begin{pmatrix} G_E J_{EE}\,u_{EE}^{ss}\,x_{EE}^{ss} & -G_E J_{EI}\,u_{EI}^{ss}\,x_{EI}^{ss} \\ G_I J_{IE}\,u_{IE}^{ss}\,x_{IE}^{ss} & -G_I J_{II}\,u_{II}^{ss}\,x_{II}^{ss} \end{pmatrix}$

Steady state values and connectivity matrices of Static-RNN-type and STP-RNN models, in supra-threshold mode. For sub-threshold modes, $G_j = 0$ (see equation (2)). Note j and $i \in \{E, I\}$, $A_j \in \{E_r, I_r\}$, and j is the index of the presynaptic population, where $A_E = E_r$ and $A_I = I_r$.

Supplementary References

1 Tsodyks, M. V., Skaggs, W. E., Sejnowski, T. J. & McNaughton, B. L. Paradoxical effects of external modulation of inhibitory interneurons. *J Neurosci.* **17**, 4382-4388 (1997).

2 Wilson, H. R. & Cowan, J. D. Excitatory and inhibitory interactions in localized populations of model neurons. *Biophys J.* **12**, 1-24 (1972).

3 Ermentrout, B. Neural networks as spatio-temporal pattern-forming systems. *Reports on Progress in Physics.* **61**, 353-430 (1998).

4 Shriki, O., Hansel, D. & Sompolinsky, H. Rate models for conductance-based cortical neuronal networks. *Neural Comput.* **15**, 1809-1841 (2003).

5 Tsodyks, M., Pawelzik, K. & Markram, H. Neural networks with dynamic synapses. *Neural Comput.* **10**, 821-835 (1998).

6 Zucker, R. S. & Regehr, W. G. Short-term synaptic plasticity. *Annu Rev Physiol.* **64**, 355-405 (2002).

7 Murphy, B. K. & Miller, K. D. Balanced amplification: a new mechanism of selective amplification of neural activity patterns. *Neuron.* **61**, 635-648 (2009).

8 Ozeki, H., Finn, I. M., Schaffer, E. S., Miller, K. D. & Ferster, D. Inhibitory stabilization of the cortical network underlies visual surround suppression. *Neuron.* **62**, 578-592 (2009).

9 Barak, O. & Tsodyks, M. Persistent activity in neural networks with dynamic synapses. *PLoS Comput Biol.* **3**, e35; 10.1371/journal.pcbi.0030035 (2007).

10 Abbott, L. F., Varela, J. A., Sen, K. & Nelson, S. B. Synaptic depression and cortical gain control. *Science.* **275**, 220-224 (1997).

11 Latham, P. E. & Nirenberg, S. Computing and stability in cortical networks. *Neural Comput.* **16**, 1385-1412 (2004).

12 Loebel, A. & Tsodyks, M. Computation by ensemble synchronization in recurrent networks with synaptic depression. *J Comput Neurosci.* **13**, 111-124 (2002).

13 Ben-Ari, Y., Gaiarsa, J. L., Tyzio, R. & Khazipov, R. GABA: a pioneer transmitter that excites immature neurons and generates primitive oscillations. *Physiol Rev.* **87**, 1215-1284 (2007).

14 Etherington, S. J. & Williams, S. R. Postnatal development of intrinsic and synaptic properties transforms signaling in the layer 5 excitatory neural network of the visual cortex. *J Neurosci.* **31**, 9526-9537 (2011).

15 Golshani, P. *et al.* Internally mediated developmental desynchronization of neocortical network activity. *J Neurosci.* **29**, 10890-10899 (2009).

16 Rochefort, N. L. *et al.* Sparsification of neuronal activity in the visual cortex at eye-opening. *Proc Natl Acad Sci USA.* **106**, 15049-15054 (2009).

17 Colonnese, M. T. *et al.* A conserved switch in sensory processing prepares developing neocortex for vision. *Neuron.* **67**, 480-498 (2010).

18 Feldmeyer, D. & Radnikow, G. Developmental alterations in the functional properties of excitatory neocortical synapses. *Journal of Physiology-London.* **587**, 1889-1896 (2009).

19 Ma, Y., Hu, H. & Agmon, A. Short-term plasticity of unitary inhibitory-to-inhibitory synapses depends on the presynaptic interneuron subtype. *J Neurosci.* **32**, 983-988 (2012).

20 Cheetham, C. E. J. & Fox, K. Presynaptic Development at L4 to L2/3 Excitatory Synapses Follows Different Time Courses in Visual and Somatosensory Cortex. *J Neurosci.* **30**, 12566-12571 (2010).

21 Tang, A. H., Chai, Z. & Wang, S. Q. Dark rearing alters the short-term synaptic plasticity in visual cortex. *Neurosci Lett.* **422**, 49-53 (2007).

22 Varela, J. A., Song, S., Turrigiano, G. G. & Nelson, S. B. Differential depression at excitatory and inhibitory synapses in visual cortex. *J Neurosci.* **19**, 4293-4304 (1999).
23 Kirmse, K., Witte, O. W. & Holthoff, K. GABAergic depolarization during early cortical development and implications for anticonvulsive therapy in neonates. *Epilepsia.* **52**, 1532-1543 (2011).
24 Levene, I. M. & Chervenak, A. F. *Fetal and Neonatal Neurology and Neurosurgery*. (Elsevier Churchill Livingstone, 2008).
25 De Felipe, J., Marco, P., Fairen, A. & Jones, E. G. Inhibitory synaptogenesis in mouse somatosensory cortex. *Cereb Cortex.* **7**, 619-634 (1997).
26 Kirmse, K. *et al.* GABA depolarizes immature neurons and inhibits network activity in the neonatal neocortex in vivo. *Nat. Commun.* **6**; 10.1038/ncomms8750 (2015).
27 Valeeva, G., Tressard, T., Mukhtarov, M., Baude, A. & Khazipov, R. An Optogenetic Approach for Investigation of Excitatory and Inhibitory Network GABA Actions in Mice Expressing Channelrhodopsin-2 in GABAergic Neurons. *J Neurosci.* **36**, 5961-5973 (2016).
28 Izhikevich, E. *Dynamical systems in neuroscience : the geometry of excitability and bursting*. Computational neuroscience (MIT Press, 2007).
29 Tsodyks, M., Uziel, A. & Markram, H. Synchrony generation in recurrent networks with frequency-dependent synapses. *J Neurosci.* **20**, 1-5 (2000).
30 Prida, L. M. & Sanchez-Andres, J. V. Nonlinear frequency-dependent synchronization in the developing hippocampus. *J Neurophysiol.* **82**, 202-208 (1999).
31 Shu, Y., Hasenstaub, A. & McCormick, D. A. Turning on and off recurrent balanced cortical activity. *Nature.* **423**, 288-293 (2003).
